# Intelligent automated essay scoring under uncertainty using type 2 neutrosophic ontologies

**DOI:** 10.1038/s41598-026-54596-9

**Published:** 2026-06-03

**Authors:** Saad M. Darwish, Noha A. Bagi, Noha A. El-Shoafy

**Affiliations:** 1 Department of Information Technology, Institute of Graduate Studies and Research, Alexandria University, 163 Horreya Avenue, El-Shatby, Alexandria, 21526 Egypt; 2 Computer Science Department, Faculty of Computers and Artificial Intelligence, Matrouh University, Matrouh, 51511 Egypt

**Keywords:** Uncertainty-Aware Computing, Type-2 Neutrosophic Logic, Soft Computing under Uncertainty, Ontology-Based Uncertain Reasoning, Uncertain Information Modeling, Engineering, Mathematics and computing

## Abstract

Computing with uncertainty has become essential across numerous domains, including decision support systems, natural language understanding, and intelligent assessment, where incomplete, imprecise, or conflicting information often arises. In the field of education, one such challenge appears in automated essay scoring (AES), where linguistic ambiguity, subjective interpretation, and vague expressions introduce significant uncertainty. Traditional ontology-based AES systems attempt to structure domain knowledge to evaluate student essays, but they often fall short in handling these uncertainties effectively, resulting in reduced scoring reliability. To address this, we propose a novel approach that integrates Type-2 Neutrosophic Sets (T2NS) into ontology-driven AES frameworks. This integration enables a more refined representation of uncertain, indeterminate, and inconsistent information in essay evaluation. Our method involves concept extraction, rule-based scoring via ontologies, the extension of the ontology using T2NS, and the application of membership functions to quantify varying levels of uncertainty. Experiments conducted on benchmark AES datasets reveal that our T2NS-enhanced framework significantly outperforms conventional methods, offering more robust and interpretable scoring decisions under uncertainty.

**Supplementary Information:**

The online version contains supplementary material available at 10.1038/s41598-026-54596-9.

## Introduction

Uncertainty associated with different domains often hinders decision-making, and similar challenges are evident in the current landscape of AES, which has become a dominant technique in intelligent learning systems^1,2^. Despite its efficiency, scalability, and ability to deliver consistent evaluations compared to manual grading, AES struggles with key sources of ambiguity, such as the interpretation of polysemous words, syntactic complexities, and contextual variations that directly impact scoring accuracy. Moreover, the reliance on human-annotated data introduces inherent variability and bias, complicating the modeling of fair and objective scoring criteria^3^.

To address these issues, AES has evolved through several methodological categories: rule-based systems emphasizing grammar and syntax analysis, statistical approaches utilizing machine learning to detect patterns in annotated essays, natural language processing (NLP) techniques designed to assess coherence and cohesion, knowledge-based systems integrating domain-specific ontologies for conceptual alignment, and hybrid models that strategically combine multiple techniques to enhance overall reliability and performance^4–6^.

Uncertainty in ontology-based AES primarily stems from the difficulty of capturing the nuanced meanings, contextual dependencies, and implicit knowledge embedded in student writing^7^. Unlike surface-level statistical methods (essay length, average word frequency, sentence complexity, lexical diversity, grammar error counts, and usage of particular keywords or n-gram), ontology-based AES incorporates structured domain knowledge, enabling semantic interpretation of essays by mapping words and phrases to well-defined concepts and their interrelationships^8,9^. This semantic layer allows the system to evaluate not only linguistic correctness but also the depth of understanding, logical flow of ideas, and conceptual alignment with domain-specific knowledge^10^.

To mitigate ambiguity and vagueness, advanced reasoning techniques such as fuzzy logic and neutrosophic sets are applied, supporting more robust decision-making when confronted with incomplete or uncertain information. Neutrosophic ontology, in particular, enriches AES by representing content with degrees of truth, falsity, and indeterminacy, thereby accommodating subjectivity and multiple valid interpretations. Such integration enhances adaptability across diverse essay genres, including argumentative, reflective, and technical compositions, while also improving fairness, reducing bias, and ensuring more context-sensitive evaluations^11–13^.

Type-2 neutrosophic ontology represents an important advancement over traditional (Type-1) neutrosophic ontology by introducing an additional dimension of uncertainty, which is highly relevant for AES. While Type-1 Neutrosophic Logic (T1NL) models uncertainty through three membership functions—truth, indeterminacy, and falsity—it is limited to a single level of representation, capturing only the direct uncertainty in membership values. In contrast, Type-2 Neutrosophic Logic (T2NL) extends this framework by incorporating the Footprint of Uncertainty (FOU), which accounts not only for the degree of membership but also for uncertainty in the membership function itself^14,15^.

This second layer enables T2NL to represent “uncertainty about uncertainty,” a critical capability when dealing with ambiguous or imprecise essay content where conceptual boundaries are unclear or overlapping. For AES, this distinction is significant: whereas T1NL can address basic indeterminacy in interpreting essay features, T2NL provides a richer and more flexible model for handling complex linguistic ambiguity, subjective judgments, and nuanced evaluation criteria. By capturing these multi-level uncertainties, T2NL enhances the robustness, adaptability, and accuracy of ontology-based AES systems, particularly in contexts that demand fine-grained and context-sensitive assessment. Table 1 provides a comparative overview of uncertainty-based ontology approaches in the context of AES.

While fuzzy ontology and Type-1 neutrosophic ontology have demonstrated effectiveness in handling basic vagueness and subjectivity in AES, their practical scoring behavior remains constrained by fixed or single-level uncertainty representations. For example, in an argumentative essay discussing climate change mitigation, a student may partially explain “carbon neutrality” while ambiguously linking it to economic policy. A fuzzy ontology-based AES system typically assigns a single membership value reflecting partial correctness, without distinguishing whether the ambiguity arises from incomplete conceptual understanding or merely imprecise linguistic expression, thereby collapsing qualitatively different grading situations into the same numeric score.

Similarly, a Type-1 neutrosophic ontology may assign moderate truth and indeterminacy values to the concept, yet these values are treated as precise and certain, preventing the model from representing evaluator hesitation or the existence of multiple plausible interpretations of the same response. In practice, however, evaluators are often uncertain about how confidently such truth and indeterminacy levels should be assigned, especially when multiple interpretations of the same passage are plausible, which restricts the system’s ability to faithfully model real grading behavior observed in human assessment.

The proposed Type-2 neutrosophic ontology-based AES framework advances beyond these limitations by explicitly modeling uncertainty in the membership functions through FOU. Consider a reflective essay in which a student uses metaphorical language to describe “learning as a journey,” partially aligning with pedagogical concepts but lacking explicit theoretical grounding. Rather than producing a single semantic relevance score, the proposed framework generates an uncertainty-aware score range that reflects varying interpretations of the metaphorical expression. This enables the AES system to indicate not only the degree of conceptual alignment but also the confidence associated with that judgment. Such confidence-aware scoring closely mirrors human grading practices, where instructors often hesitate between adjacent score levels due to ambiguous phrasing. As a result, the proposed approach significantly enhances interpretability by making uncertainty explicit and traceable in grading decisions.

**Table 1:** Comparison of Fuzzy Ontology, Type-1 Neutrosophic Ontology, and Type-2 Neutrosophic Ontology in AES.

Aspect	Fuzzy Ontology	Type-1 Neutrosophic Ontology	Type-2 Neutrosophic Ontology
**Uncertainty Representation**	Captures vagueness with degrees of membership between 0 and 1	Represents uncertainty with three functions: truth (T), indeterminacy (I), and falsity (F)	Adds a second layer of uncertainty, handling not only T, I, F values but also uncertainty about these values
**Handling** **Ambiguity**	Limited to approximate reasoning	Better handling of ambiguity by separating truth, falsity, and indeterminacy	Superior handling of complex and overlapping ambiguities through FOU
**Suitability for AES**	Effective for basic grading tasks involving vague linguistic features	Suitable for modeling essay content with subjective interpretations	Highly effective for essays with complex semantics, overlapping concepts, and unclear evaluation criteria
**Strengths**	Simplicity and computational efficiency	Balanced treatment of different types of uncertainty	Comprehensive representation of multi-level uncertainty; robustness in diverse essay types
**Limitations**	Cannot explicitly model indeterminacy	Limited to single-level uncertainty	Increased computational complexity and modeling overhead
**Application in AES**	Useful for grammar checks, word similarity, and stylistic features	Effective in evaluating coherence, conceptual relevance, and subjective judgments	Suitable for advanced AES tasks requiring fine-grained, context-sensitive, and multi-perspective evaluation

### Problem statement

Uncertainty is a central challenge in semantic-based AES systems, as essays often contain ambiguous, vague, or context-dependent language that is difficult to interpret reliably. Words and phrases may carry multiple meanings, and distinguishing between literal, figurative, or domain-specific usage introduces significant semantic ambiguity. This uncertainty prevents AES systems from fully capturing the depth of reasoning, coherence, and conceptual nuances present in student writing. While ontology integration has emerged as a promising strategy to reduce ambiguity and enhance semantic understanding, its effectiveness depends heavily on the completeness and precision of the ontology. Missing concepts, incomplete relationships, or insufficient semantic coverage can further propagate uncertainty, resulting in partial interpretations and inaccurate scoring. Therefore, addressing uncertainty in semantic analysis remains a critical problem for advancing AES toward more reliable and context-sensitive evaluation.

### Motivation of the work

Neutrosophic ontology-based automated essay scoring (NOAES) represents an innovative approach that combines neutrosophic logic with ontology to address uncertainties and ambiguities in AES such as vague language, subjective interpretations, and ambiguous expressions. Neutrosophic ontology allows for flexible grading criteria, where the degree of truth, falsity, and indeterminacy associated with each grading criterion can be explicitly defined. These systems still faces challenges in effectively managing higher levels of uncertainty. Addressing this challenge requires interdisciplinary research efforts at the intersection of neutrosophic logic, ontology engineering, natural language processing, and machine learning.

Developing robust frameworks that can effectively handle higher levels of uncertainty is essential for advancing the state-of-the-art in neutrosophic ontology-based AES. The adoption of type-2 neutrosophic ontology in AES can hold promise for effectively handling higher levels of uncertainty in essay scoring. By providing a more refined and flexible framework for uncertainty modeling and reasoning, type-2 neutrosophic ontology can enhance the reliability, interpretability, and scalability of AES systems, ultimately leading to more accurate and trustworthy scoring outcomes.

### Contribution and methodology

This paper builds upon the work in Ref^13^., which employed traditional (Type-1) neutrosophic ontology within an AES framework, by extending it to a Type-2 neutrosophic formulation for enhanced uncertainty modeling. The primary contribution lies in upgrading the representation of uncertainty from fixed membership degrees to Type-2 neutrosophic sets, where truth, indeterminacy, and falsity are characterized using FOU. This extension enables the framework to capture higher-order uncertainty associated with ambiguity, vagueness, semantic overlap, and context-dependent variation in student writing.

Within the proposed system, ontology-driven reasoning and Type-2 neutrosophic modeling constitute the core components responsible for inference and score interpretation. In contrast, techniques such as TF-IDF, Word2Vec, BERT embeddings, and spatial autocorrelation metrics serve as supporting modules for feature extraction and semantic representation, and can be adapted without affecting the fundamental contribution. By explicitly modeling uncertainty at a higher level, the proposed approach improves robustness, interpretability, and fairness in scoring, particularly in cases where student responses exhibit partial correctness or linguistic imprecision. As shown in Table 2, the proposed framework is structured into core modules that define the main methodological contribution, and auxiliary modules that support feature extraction and semantic enhancement without affecting the underlying uncertainty modeling mechanism.

### Research questions

To guide this investigation, the following research questions are formulated:

#### RQ1

How can Type-2 neutrosophic ontology be effectively integrated into Automated Essay Scoring frameworks to model and manage higher-order uncertainty?

#### RQ2

To what extent does the proposed Type-2 neutrosophic ontology-based AES model improve the handling of ambiguity, vagueness, and semantic overlap compared to traditional statistical and Type-1 ontology-based approaches?

#### RQ3

Can the adaptive scoring criteria enabled by Type-2 neutrosophic ontology enhance fairness, transparency, and reliability in essay evaluations across diverse writing styles and genres?

#### RQ4

What is the comparative performance of the proposed framework on benchmark AES datasets, and how does it advance the state-of-the-art in scoring accuracy and interpretability under uncertain conditions?

**Table 2:** Core and Auxiliary Components of the Proposed AES Framework.

Category	Module/ Technique	Role in the Framework	Contribution to AES System
**Core Modules**	Ontology-Based Reasoning	Provides structured representation of domain knowledge and supports rule-based inference over essay content.	Enables semantic alignment between student responses and expected concepts for scoring.
**Core Modules**	Type-2 Neutrosophic Uncertainty Modeling	Extends traditional neutrosophic ontology by modeling truth, indeterminacy, and falsity using FOU.	Handles higher-order uncertainty, ambiguity, and contextual variation in student writing; forms the primary novelty of the framework.
**Auxiliary** **Modules**	TF-IDF	Extracts statistical term importance from essay texts.	Supports lexical-level feature representation for downstream semantic processing.
**Auxiliary** **Modules**	Word2Vec Embeddings	Captures distributed semantic relationships between words.	Enhances semantic similarity estimation between student answers and reference concepts.
**Auxiliary** **Modules**	BERT Embeddings	Provides contextualized deep semantic representations of text.	Improves understanding of contextual meaning and disambiguation in student essays.
**Auxiliary** **Modules**	Spatial Autocorrelation Metrics	Measures structural and distributional patterns in feature space.	Assists in analyzing coherence and consistency of extracted features across responses.

The remainder of this paper is organized as follows. Section 2 reviews the existing literature on essay evaluation systems, highlighting key methodologies and their limitations. Section 3 introduces the proposed AES model based on Type-2 neutrosophic ontology and describes its underlying framework. Section 4 presents the experimental evaluation of the proposed model, including the results and an in-depth discussion of findings. Finally, Sect. 5 concludes the study and outlines potential directions for future research.

## Related work

AES has evolved along multiple research directions, including statistical and deep learning models, ontology-driven symbolic systems, and uncertainty-aware knowledge representations. Recent surveys confirm the rapid progress of neural approaches while also emphasizing persistent concerns regarding interpretability, fairness, and robustness^2,4,16,17,45^.

### Deep neural AES

Deep neural networks (DNNs) and transformer-based models have achieved strong predictive accuracy by learning rich semantic and syntactic representations directly from text. Comprehensive reviews of these models are provided in^16,17,45^. Despite their effectiveness, several limitations remain. First, DNNs typically require large volumes of expert-scored essays, making adaptation to new prompts costly. Second, they may generalize poorly to unseen topics or writing styles, leading to unstable or biased outcomes. Third, their decision processes are often opaque, complicating justification of scores in high-stakes educational contexts. Studies on validity and fairness repeatedly highlight the need for more transparent and controllable mechanisms^34,37,38^. These challenges have motivated interest in approaches that encode knowledge explicitly rather than implicitly through parameters.

### Ontology and symbolic AES

Ontology-based AES relies on structured domain knowledge, semantic relations, and rule reasoning. Because evaluation criteria are represented explicitly, such systems often provide greater interpretability and can function with limited labeled data^18–20^. Ajetunmobi and Daramola^7^ employed ontology-guided information extraction to match essay content with subject concepts. Darwish and Mohamed^8^ fused fuzzy ontology with latent semantic analysis to enhance semantic coverage. Contreras et al. proposed ontology-supported grading pipelines combining ontology construction with NLP processing^9,23^. Other efforts have aimed at building fully integrated e-assessment environments^10^ or subject-focused systems whose outputs correlate well with expert judgments^21^.

Beyond essay scoring, ontology-driven methods have been applied to automated question generation^24,25^, multiple-choice evaluation without model answers^26^, keyword and topic identification^27^, exam coverage analysis^28^, and collaborative learning analytics^29^. These studies collectively demonstrate the strength of ontologies in structuring knowledge, enabling traceability, and reducing computational demands. However, classical ontologies are crisp. They assume that a concept either holds or does not hold, which is often unrealistic for natural language interpretation.

Although automated question generation and multiple-choice assessment differ from essay scoring in terms of evaluation granularity and output structure, these studies remain relevant to the present work because they illustrate the broader role of ontologies in educational assessment systems. In particular, ontology-based question generation and evaluation frameworks demonstrate how domain knowledge can be formally modeled, reused across assessment tasks, and reasoned over to support automation, scalability, and traceability. These properties are equally critical in automated essay scoring, where domain concepts, relationships, and evaluation criteria must be explicitly represented and systematically matched against student responses. Consequently, such works are cited not as direct AES solutions, but as evidence of the maturity and flexibility of ontology-driven assessment infrastructures upon which the proposed framework builds.

### Fuzzy and neutrosophic extensions

To better represent vagueness, researchers introduced fuzzy ontologies, allowing graded membership. This strategy improves flexibility in interpreting partial matches and imprecise statements. Nevertheless, the definition of linguistic variables and membership functions is frequently subjective, and different experts may construct different models. Neutrosophic ontologies further extend this idea by treating truth, falsity, and indeterminacy as independent components. Applications have appeared in domains such as competence modeling^11^ and medical data classification^12^. Within AES, Darwish et al.^13^ demonstrated that neutrosophic reasoning can enhance interpretability and better reflect ambiguity in students’ responses. Yet, most existing implementations rely on Type-1 neutrosophic representations, where the degrees themselves are assumed to be precise.

In real essay evaluation, evidence supporting a concept is rarely stable. A passage may partially satisfy a criterion, conflict with another, or remain open to interpretation depending on linguistic nuance. Fuzzy systems capture gradual satisfaction but do not explicitly separate contradiction from indeterminacy. Neural models may learn such patterns implicitly but cannot expose or control them. Therefore, there remains a need for an AES framework that: preserves the interpretability of ontology reasoning, models vagueness, distinguishes truth, falsity, and indeterminacy, and represents uncertainty about those assessments themselves. To the best of our knowledge, this combination has not been realized within AES. The present work addresses this gap by integrating Type-2 neutrosophic sets into an ontology-driven scoring pipeline, enabling more faithful modeling of ambiguous and conflicting linguistic evidence. As shown in Table 3, no previous AES framework simultaneously combines ontology-based interpretability with explicit indeterminacy modeling and Type-2 uncertainty representation.

**Table 3:** Summarizes the capability differences between existing paradigms and the proposed framework.

Approach/Representative Works	Interpretability	Handles Vagueness (graded truth)	Represents Indeterminacy explicitly	Models Uncertainty in Membership (Type-2)	Applied to AES
Deep Neural AES^16,17,45^	Low	Implicit	✖	✖	✔
Neural AES with validity/measurement analysis^34,37,38^	Low–Medium	Implicit	✖	✖	✔
Classical ontology-based AES^7,9,10,21,23^	High	✖	✖	✖	✔
Fuzzy ontology AES^8^	High	✔	✖	✖	✔
Type-1 neutrosophic ontology AES^13^	High	✔	✔	✖	✔
Type-2 neutrosophic applications in other domains^14,15,30,31^	High	✔	✔	✔	✖
**Proposed framework**	**High**	✔	✔	✔	✔

### Research gap

Despite significant progress in AES, existing approaches still face major limitations when dealing with uncertainty in student writing. Deep neural network–based models, while powerful, require large labeled datasets, high computational resources, and often suffer from poor interpretability, bias, and weak generalization across diverse writing styles^16^. Ontology-based methods offer greater transparency and data efficiency, yet their effectiveness depends heavily on the completeness and accuracy of the constructed ontology.

Designing such ontologies demands extensive domain expertise and remains vulnerable to subjective choices in defining concepts, relationships, and evaluation criteria^17–20^. Furthermore, ontology-driven AES must rely on sophisticated NLP techniques to extract semantic information, a task complicated by variability in writing styles, vocabulary, and conventions. The use of fuzzy ontology has improved handling of vagueness by allowing partial membership, but its reliance on subjectively defined linguistic variables, membership functions, and fuzzy rules still introduces inconsistencies across implementations.

In this context, neutrosophic ontology has emerged as a promising framework, as it extends fuzzy ontology to explicitly model truth, indeterminacy, and falsity, thereby offering a richer representation of uncertainty^13^. However, current neutrosophic ontology–based AES approaches are limited to Type-1 representations, which can only capture single-layer uncertainty. They fall short in addressing higher-order uncertainty, such as situations where even the degree of indeterminacy is uncertain. This leaves a critical gap in ensuring robust, reliable, and context-sensitive scoring.

To address this gap, this paper proposes a Type-2 Neutrosophic Ontology-based AES framework. By incorporating the FOU, the system enables modeling of both primary uncertainty and uncertainty about uncertainty, offering a more nuanced and expressive framework. This approach directly tackles the challenges of ambiguity, vagueness, and semantic variability identified in prior works, with the goal of producing more accurate, reliable, and fair assessments of student writing. The following section presents the proposed framework in detail.

## Methodology

### Conceptual overview of the T2NO-AES framework

In the proposed model, automated essay scoring is enhanced through a T2NO that explicitly models uncertainty and ambiguity inherent in essay evaluation. The ontology encodes domain-specific concepts, relationships, and axioms, forming a structured knowledge base for reasoning about essay content, coherence, and relevance. Essay features are evaluated against this ontology, while the Type-2 neutrosophic framework represents the degrees of truth, indeterminacy, and falsity associated with each concept and relationship using interval-valued memberships at two hierarchical layers.

By incorporating FOU, the model captures both lower and upper bounds of uncertainty, enabling a nuanced interpretation of essay quality even when content is vague, contradictory, or only partially aligned with expected domain knowledge. This uncertainty-aware reasoning allows the system to make informed scoring decisions in situations where traditional automated scoring approaches often struggle. As a result, the T2NO-basedAES framework provides a robust and flexible mechanism for mapping essay features to appropriate scores while systematically accounting for uncertainty^7–10,21,22^.

The proposed framework follows a structured processing pipeline adapted from ontology-based AES approaches^7–9^. Essays are first preprocessed to clean and normalize the text, remove irrelevant elements, and standardize formatting for semantic analysis. Linguistic, structural, and ontology-based semantic features are then extracted and represented numerically. Using NLP techniques guided by the ontology, relevant concepts and relationships are identified, and semantic coherence and content relevance are assessed. A domain-specific ontology is developed to define the concepts, relationships, and constraints required for essay evaluation and is integrated into the system through a Type-2 neutrosophic knowledge representation framework.

Machine-learning models are subsequently trained on labeled essays using the extracted features to predict scores while capturing both traditional linguistic patterns and ontology-encoded semantic knowledge. Model performance is evaluated on separate test data using standard evaluation metrics such as accuracy and correlation coefficients. Once validated, the trained model is deployed to automatically score new essays using the same ontology-driven semantic analysis pipeline.

**Fig. 1:**
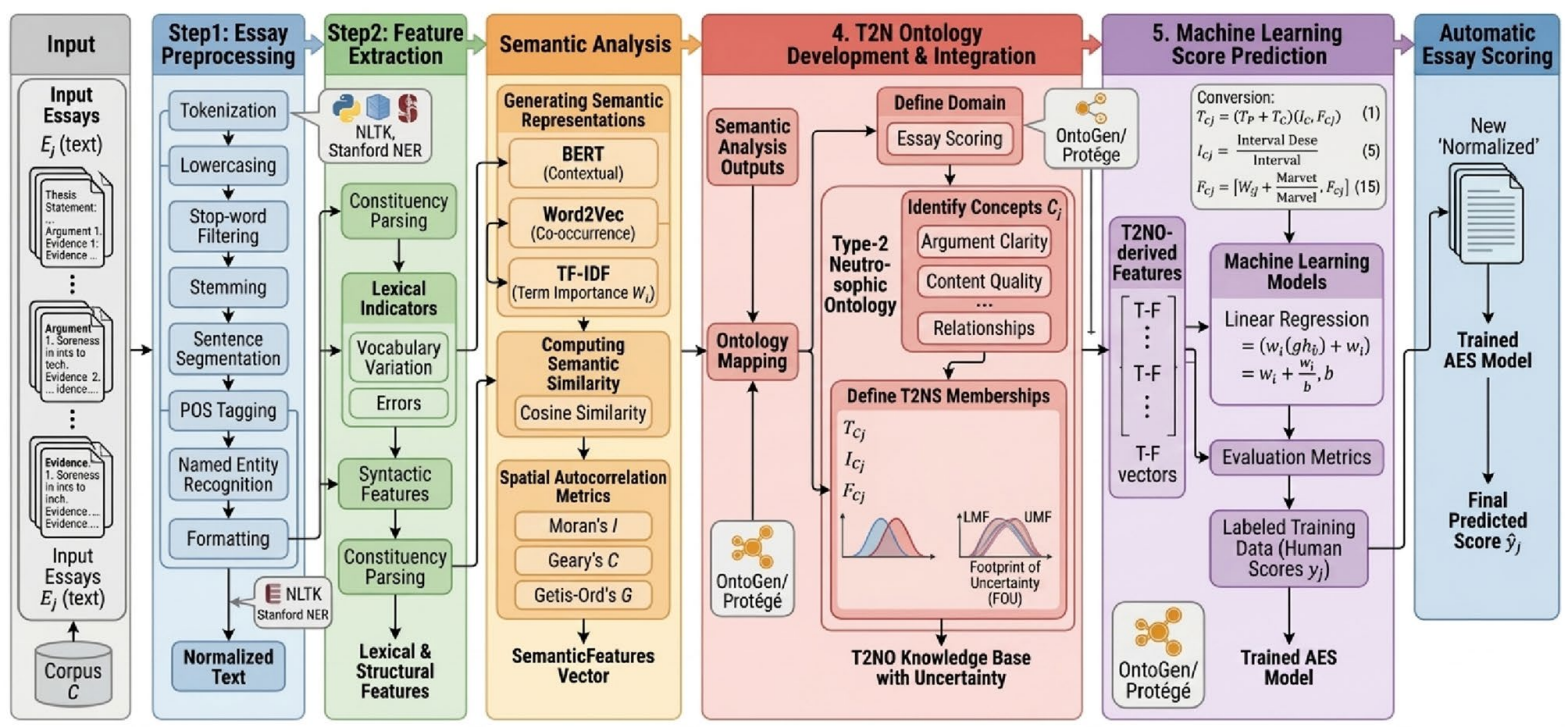
High-Level Overview of the Proposed T2NO-based Essay Scoring Model.

Figure 1 illustrates the overall architecture of the proposed automated essay scoring system, highlighting the sequential flow from preprocessing and feature extraction to ontology-based reasoning, model training, evaluation, and final score generation. Table 4 summarizes the key mathematical notations used throughout the formulation of the Type-2 Neutrosophic Ontology-based automated essay scoring framework.

**Table 4:** Summary of Notations Used in the T2NO-Based AES Framework.

Symbol/Notation	Description
$\:E$	Input essay text
$\:P$	Essay prompt or expected response
$\:\mathcal{D}$	Corpus (collection of essays)
$\:C$	Ontology concept (e.g., argument clarity, content quality)
$\:{f}_{c}$	Normalized feature value associated with concept $\:C$
$\:{f}_{c}^{\left(i\right)}$	Feature value of concept $\:C$ for the $\:i$th essay
$\:N$	Total number of essays in the training corpus
$\:{\mu\:}_{c}$	Mean of feature $\:{f}_{c}$ across the corpus
$\:{\sigma\:}_{c}$	Standard deviation of feature $\:{f}_{c}$
$\:{T}_{C}$	Truth-membership interval of concept $\:C$
$\:{T}_{C}^{min},{T}_{C}^{max}$	Lower and upper bounds of truth membership
$\:{I}_{C}$	Indeterminacy-membership interval of concept $\:C$
$\:{I}_{C}^{min},{I}_{C}^{max}$	Lower and upper bounds of indeterminacy
$\:{F}_{C}$	Falsity-membership interval of concept CCC
$\:{F}_{C}^{min},{F}_{C}^{max}$	Lower and upper bounds of falsity
$\:\alpha\:$	Scaling parameter controlling indeterminacy expansion
$\:{FOU}_{C}$	Footprint of Uncertainty for concept $\:C$
$\:{W}_{T}$	Width of truth interval$\:\:({T}_{C}^{max}-{T}_{C}^{min})$
$\:{W}_{I}$	Width of indeterminacy interval
$\:{W}_{F}$	Width of falsity interval
$\:TF$	Term Frequency of a word in an essay
$\:IDF$	Inverse Document Frequency across corpus
$\:TF-IDF$	Weighted importance of a term
$\:{\mathbf{v}}_{\boldsymbol{i}}$	Vector representation of sentence/word embedding
$\:cos\left(\theta\:\right)$	Cosine similarity between semantic vectors
$\:BERT$	Contextual embedding model for semantic representation
$\:Word2Vec$	Embedding model capturing word co-occurrence patterns
$\:Moran's\:I$	Global spatial autocorrelation (semantic clustering)
$\:Geary's\:C$	Local variation in semantic space
$\:Getis-Ord\:G$	Local clustering intensity of semantic concepts
**x**	Feature vector derived from ontology (e.g., T − F)
$\:{x}_{j}$	$\:j$-th feature in vector **x**
$\:\widehat{y}$	Predicted essay score
$\:y$	Actual (human-assigned) score
$\:{\beta\:}_{0}$	Bias term in regression model
$\:{\beta\:}_{j}$	Weight for feature $\:{x}_{j}$
$\:\lambda\:$	Regularization parameter (Ridge/Lasso)

### Implementation details

**Step 1: Essay Preprocessing**.

Preprocessing prepares essays for subsequent semantic and ontology-based analysis by converting raw textual input into a consistent linguistic format. The procedure includes tokenization, lowercasing, punctuation removal, stop-word filtering, stemming or lemmatization, sentence segmentation, part-of-speech tagging, named entity recognition, and elimination of non-content elements such as HTML tags, citations, headers, and special characters. These operations ensure that essays are represented in a normalized structure suitable for downstream processing.

The preprocessing stage also reduces variability introduced by inconsistent formatting, lexical inflections, and irrelevant textual artifacts. By standardizing the textual input, the framework enables more reliable extraction of lexical, syntactic, and semantic patterns that later contribute to ontology population and uncertainty-aware scoring. Common NLP libraries such as NLTK, Stanford NER, and Python-based text-cleaning utilities are used to implement these operations.

**Step 2: Feature Extraction and Representation**.

Following preprocessing, essays are transformed into structured representations through lexical and syntactic feature extraction. Constituency parsing is employed to capture sentence structure by identifying hierarchical phrase relationships, enabling analysis of grammatical organization. Lexical characteristics are then computed to represent vocabulary richness, linguistic correctness, and informational density.

The extracted lexical indicators include vocabulary variation with and without errors, lexical error percentage, and lexical density. Together, these measures provide a multidimensional description of writing quality by quantifying vocabulary diversity, lexical accuracy, and information-bearing content. The resulting feature representations form a numerical basis for subsequent semantic interpretation and ontology mapping.

**Step 3: Semantic Analysis**.

Semantic analysis evaluates conceptual organization, topical relevance, and coherence across essay content. The process identifies dominant themes, measures alignment between essay content and prompts, and examines continuity between ideas. To represent semantic information numerically, TF-IDF weighting, contextual embeddings, and semantic similarity measures are applied to generate vector-based representations of essay content.

Semantic relationships are further characterized through cosine similarity and spatial autocorrelation metrics, including Moran’s I, Geary’s C, and Getis-Ord’s G. These measures provide information about conceptual proximity, local variation, and clustering patterns within semantic embedding spaces. The resulting semantic features are aggregated into a unified representation that supports ontology construction and uncertainty-aware reasoning^33,34^.

#### Algorithm 1

summarizes the semantic analysis process for essays, integrating feature extraction, vector-based representations, cosine similarity, and spatial autocorrelation metrics to evaluate coherence, relevance, and topical focus.

**Figure:**
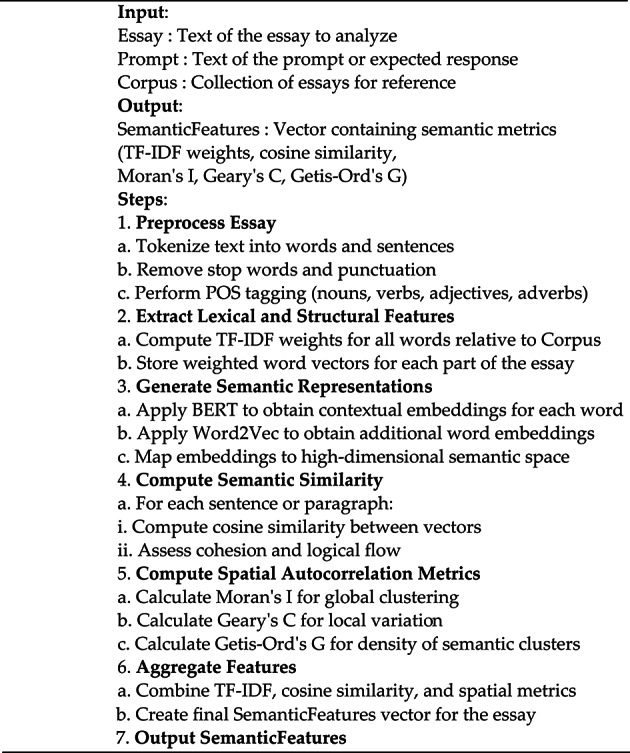
Algorithm 1: SemanticAnalysis(Essay, Prompt, Corpus).

### Multi-level semantic representations for ontology construction

The proposed framework integrates multiple semantic representations to support ontology construction and uncertainty modeling within the essay scoring process. TF-IDF contributes information regarding term importance relative to the corpus, enabling the identification of domain-relevant concepts that serve as ontology candidates. Word2Vec captures semantic proximity among terms based on contextual co-occurrence patterns, facilitating the discovery of relationships and conceptual groupings. BERT embeddings provide context-sensitive representations that preserve sentence-level meaning and assist in distinguishing concept usage across varying linguistic contexts.

Rather than functioning as independent scoring mechanisms, these semantic representations operate as complementary inputs to the ontology layer. Their outputs are combined into a unified feature space that guides concept creation, relationship definition, and attribute assignment within the Type-2 neutrosophic ontology. This integration allows semantic evidence extracted from essays to be translated into interval-valued truth, indeterminacy, and falsity memberships associated with ontology concepts.

The ontology therefore acts as the central reasoning structure that transforms semantic representations into uncertainty-aware knowledge. Features extracted from TF-IDF, Word2Vec, and BERT are not used solely for similarity estimation but are incorporated into ontology nodes and relations to model ambiguity, partial correctness, and semantic overlap. Through this process, the framework links conventional NLP representations with Type-2 neutrosophic reasoning, enabling essay evaluation under varying degrees of uncertainty.

This design clarifies the distinction between supporting NLP components and the primary methodological contribution. Semantic embeddings provide descriptive representations of essay content, while the ontology layer organizes these representations into a structured knowledge model enriched with interval-based uncertainty semantics. The resulting integration strengthens interpretability and supports consistent reasoning across heterogeneous linguistic patterns.

#### Semantic feature integration within the type-2 neutrosophic ontology

The integration of semantic representations into the Type-2 neutrosophic ontology occurs through a structured mapping process in which extracted semantic indicators are aligned with ontology concepts. Each ontology concept receives evidence from multiple semantic sources, allowing the framework to associate linguistic patterns with domain-specific assessment dimensions. For example, semantic similarity scores contribute to concepts related to coherence and topic alignment, while lexical indicators support concepts linked to vocabulary quality and content richness. This mapping process ensures that semantic evidence is not treated independently but becomes part of a unified ontology-driven representation.

Within the ontology, semantic features are converted into interval-valued memberships that characterize the degree to which an essay satisfies a given evaluative concept. Rather than assigning a single deterministic value, the framework represents each concept using lower and upper bounds for truth, indeterminacy, and falsity. These intervals are derived from empirical feature distributions and semantic variability observed across essays. Consequently, the ontology can preserve uncertainty originating from ambiguous wording, partially developed arguments, or inconsistent semantic structure.

The ontology also supports hierarchical propagation of semantic evidence across related concepts. Lower-level concepts derived from lexical and semantic analysis contribute to broader assessment categories through ontology relationships. For instance, concepts such as argument clarity, evidence relevance, and semantic cohesion may collectively influence higher-level constructs such as content quality or conceptual consistency. Through this hierarchical organization, uncertainty associated with individual concepts is aggregated into broader evaluative interpretations while maintaining traceability to the originating semantic features.

A further advantage of this integration lies in the ontology’s ability to preserve contextual dependencies among semantic features. Relationships between concepts are not represented as isolated associations but as interconnected structures that reflect how multiple linguistic signals jointly contribute to essay quality. By embedding Type-2 neutrosophic intervals within these relationships, the ontology can model partial agreement, conflicting evidence, and overlapping semantic interpretations. This provides a richer representation than feature-based scoring alone, as the reasoning process considers both semantic relevance and uncertainty across interconnected concepts.

The ontology-driven integration stage therefore functions as a transition layer between feature extraction and score prediction. Semantic representations provide descriptive evidence, while the ontology organizes this evidence into a structured uncertainty-aware model suitable for reasoning. Through Type-2 neutrosophic memberships, the framework captures not only whether a concept is present but also the confidence and ambiguity associated with its interpretation. This supports a more flexible assessment process capable of handling subjective and semantically diverse essay responses.

#### Step 4: Type 2 neutrosophic ontology development and integration

Building on the semantic analysis of essays, which captures lexical richness, coherence, and semantic relationships, the next step is to formalize this information into a structured knowledge representation capable of handling uncertainty and subjectivity. Step 4 introduces Type-2 neutrosophic ontology development, which forces these semantic features to define the domain, identify relevant concepts and entities, specify the relationships between them, and capture their properties and attributes.

By integrating the high-dimensional semantic representations and clustering patterns from Step 3 into the ontology, the system can model both the objective structure of essay content and the inherent subjectivity of human judgment. The following steps detail how this ontology can be constructed based on the outputs of Step 3.

- **Define the Domain**: The first step in ontology development is defining the domain, which involves determining the specific area of knowledge that the ontology will represent—in this case, essay scoring. High-weighted keywords from TF-IDF analysis and major semantic clusters identified through cosine similarity and spatial metrics are used to determine central topics and subtopics.
- **Identify Concepts and Entities**: Next, the fundamental concepts and entities are extracted from the semantic analysis. TF-IDF highlights significant keywords, embeddings from BERT and Word2Vec capture semantic relationships, and spatial autocorrelation metrics reveal clusters of related ideas. These outputs guide the identification of core entities, such as “argument clarity,” which measures how clearly and logically ideas are presented; “evidence support,” which evaluates the extent to which claims are substantiated with examples or reasoning; and their grouping under the broader concept of “content quality,” reflecting the overall richness, relevance, and organization of the essay.
- **Define Relationships between Concepts**: Once concepts are identified, the relationships among them are specified to capture the semantic structure of essay content. Cosine similarity values indicate the closeness of concepts, while Moran’s I and Geary’s C provide insight into global and local cohesion patterns. This step ensures that the ontology reflects both the structure of ideas and their interconnections.
- **Assign Properties and Attributes**: After establishing relationships, each concept and connection is characterized with properties and attributes that reflect importance, relevance, and uncertainty. TF-IDF weights can quantify significance, cosine similarity scores and embeddings define semantic strength, and Type-2 neutrosophic values represent degrees of truth, indeterminacy, and falsity. This step ensures that the ontology captures both measurable and subjective aspects of essay quality.
- **Set Scope and Boundaries**: The scope and boundaries of the ontology are defined to maintain focus and relevance. Clusters and semantic patterns from TF-IDF, cosine similarity, and spatial autocorrelation help filter out peripheral or low-relevance concepts. This step ensures the ontology remains concise, targeted, and aligned with the intended assessment purpose.
- **Integrate Semantic Analysis Outputs into Ontology**: All outputs from Step 3 are integrated into the ontology to create a coherent knowledge structure. TF-IDF weights, embeddings, cosine similarity measures, and spatial metrics populate nodes, edges, and attributes, while neutrosophic values encode uncertainty. This integration links quantitative semantic analysis with qualitative knowledge representation.
- **Validate and Refine Ontology**: Finally, the ontology is validated to ensure accurate representation of essay knowledge and logical relationships. This step guarantees that the ontology faithfully models both the structure and uncertainty of essay content, providing a robust framework for subsequent scoring and reasoning. To operationalize Step 4, Algorithm 2 outlines how the extracted semantic features are transformed into a Type-2 neutrosophic ontology, where each concept is assigned interval-based truth, indeterminacy, and falsity memberships, along with a footprint of uncertainty that captures the inherent subjectivity of essay scoring.

**Figure:**
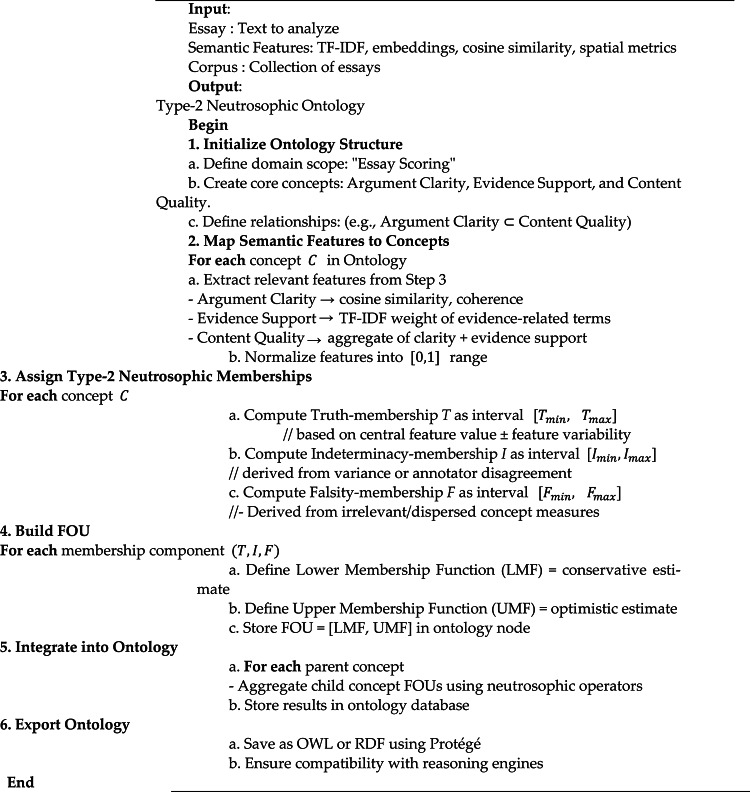
Algorithm 2: Build Type2_Neutrosophic Ontology.

### Formal definition of type-2 neutrosophic memberships and aggregation

To ensure that the interval-based neutrosophic memberships are statistically grounded rather than heuristic, the bounds of each membership component are derived from empirical feature distributions observed in the training corpus. For each evaluative feature (e.g., argument clarity, semantic coherence, or topic relevance), normalized scores are computed across benchmark essays corresponding to the same prompt.

The central tendency of these scores is represented by the mean value, while their variability is captured using the standard deviation. The lower and upper bounds of the truth membership interval are therefore defined as $\:\mu\:\:-\:\sigma\:$ and $\:\mu\:\:+\:\sigma\:$, respectively, after normalization to the $\:\left[\mathrm{0,1}\right]$ range. This formulation reflects the natural variability of essay quality within the dataset and provides an interpretable estimate of epistemic uncertainty in feature evaluation. The indeterminacy interval is derived from the observed dispersion of feature values and inter-essay variability, while the falsity component is computed as the complement of the truth interval under neutrosophic constraints.

To ensure robustness across datasets and prompts, interval widths are calibrated using corpus-specific statistics computed during the training phase, allowing the model to adapt to differences in prompt difficulty, writing styles, and scoring distributions. This statistically informed formulation ensures that the Type-2 neutrosophic intervals reflect empirical variability in essay features rather than relying on purely heuristic bounds.

let $\:{f}_{c}$ denote the normalized feature score associated with concept $\:C$ (e.g., argument clarity, topic relevance, semantic coherence) for essays belonging to the same prompt in the training corpus. Assume that feature values are normalized to the interval $\:{f}_{c}\in\:\left[\mathrm{0,1}\right]$. Let $\:{\mu\:}_{c}$ is the mean value of feature $\:{f}_{c}$ across training essays, and $\:{\sigma\:}_{c}$ is standard deviation of$\:{\:f}_{c}$. These statistics are computed as:

1 $$\:{\mu\:}_{c}=\frac{1}{N}\sum\:_{i=1}^{N}{f}_{c}^{\left(i\right)}$$

2 $$\:{\sigma\:}_{c}=\sqrt{\frac{1}{N}\sum\:_{i=1}^{N}{\left({f}_{c}^{\left(i\right)}-{\mu\:}_{c}\right)}^{2}}$$

where $\:N$ is the number of essays in the training corpus.

- **The truth membership interval** is constructed using the empirical variability of the feature:

3 $$\:{T}_{C}=[{T}_{C}^{min},{T}_{C}^{max}]$$

4 $$\:{T}_{C}^{min}=\mathrm{max}\left(0,{\mu\:}_{c}-{\sigma\:}_{c}\right)$$

5 $$\:{T}_{C}^{max}=\mathrm{min}\left(1,{\mu\:}_{c}+{\sigma\:}_{c}\right)$$

This formulation ensures that the interval remains within the normalized range $\:\left[\mathrm{0,1}\right]$. The truth interval therefore captures the expected level of feature presence with variability observed across essays.

- **Indeterminacy** reflects uncertainty due to feature variability and disagreement across essays. It is computed directly from the dispersion of the feature distribution:

6 $$\:{I}_{C}=[{I}_{C}^{min},{I}_{C}^{max}]$$

7 $$\:{I}_{C}^{min}={\sigma\:}_{c}$$

8 $$\:{I}_{C}^{max}=\mathrm{min}\left(1,{\sigma\:}_{c}+\propto\:\left(1-{\mu\:}_{c}\right)\right)$$

$\:\alpha\:\in\:\left[\mathrm{0,1}\right]$ is a scaling parameter controlling the influence of uncertainty. In practice,$\:\:\alpha\:=0.5$ provides stable uncertainty intervals. Thus, higher variance in essay features leads to larger indeterminacy intervals, reflecting increased ambiguity.

- The falsity interval represents the degree to which the concept is not supported. Under neutrosophic complementarity constraints:

9 $$\:{F}_{C}=[{F}_{C}^{min},{F}_{C}^{max}]$$

10 $$\:{F}_{C}^{min}=\mathrm{max}\left(\mathrm{0,1}-{T}_{C}^{max}\right)$$

11 $$\:{F}_{C}^{max}=\mathrm{min}\left(\mathrm{1,1}-{T}_{C}^{min}\right)$$

This ensures $\:{T}_{C}+{I}_{C}+{F}_{C}\le\:1$ and preserves the neutrosophic semantics of partial truth, uncertainty, and falsity.

The width of the neutrosophic interval quantifies the uncertainty of the membership estimate. For the truth component:

12 $$\:{W}_{T}={T}_{C}^{max}-{T}_{C}^{min}=2{\sigma\:}_{c}$$

Similarly, the widths of the other intervals are:

13 $$\:{W}_{I}={I}_{C}^{max}-{I}_{C}^{min}$$

14 $$\:{W}_{F}={F}_{C}^{max}-{F}_{C}^{min}$$

Larger interval widths indicate greater uncertainty in concept evaluation, typically caused by high variability in essay features across the training corpus.

The Type-2 neutrosophic footprint of uncertainty for concept $\:C$ is therefore defined as:

15 $$\:{FOU}_{C}=\left[\left[{T}_{C}^{min},{T}_{C}^{max}\right]\cup\:\left[{I}_{C}^{min},{I}_{C}^{max}\right]\cup\:\left[{F}_{C}^{min},{F}_{C}^{max}\right]\right]$$

This footprint represents the combined uncertainty space associated with the concept in the ontology. The width of the resulting neutrosophic intervals is directly proportional to the empirical variability of the feature distribution. Consequently, concepts exhibiting higher variability across essays produce wider intervals, reflecting greater epistemic uncertainty in the evaluation process.

All intervals $\:{T}_{c},\:{I}_{c},\:{F}_{c}\:\:$are computed relative to the specific prompt, as features such as argument strength, relevance, and coherence are normalized against prompt-specific expectations derived from benchmark essays. This ensures that the T2NS representation adapts to varying topics, essay styles, and difficulty levels.

In our case, we utilize OntoGen that is a tool developed by researchers at the University of Oxford for automatically generating ontologies from textual data. It uses machine learning techniques to analyze text corpora and extract domain-specific concepts, relationships, and axioms for ontology construction. Once the ontology is developed, integrate it with essay scoring systems. This integration can be achieved by using the ontology to define the criteria for evaluating essays.

Each criterion, such as grammar or coherence, can be mapped to specific concepts in the ontology, along with their associated type-2 neutrosophic membership degrees. Essays can then be scored by evaluating how well they adhere to each criterion according to the ontology. We can use ontology visualization tools such as Protégé with plugins or OntoVis to represent the developed type-2 neutrosophic ontology and depicts the complex relationships within the ontology.

To demonstrate the application of Type-2 Neutrosophic Ontology in automated essay scoring, we analyze a sample essay on climate change, extracting key features and converting them into a numeric vector for linear regression evaluation. The sample essay, *“Climate change is one of the most critical challenges of our time. Governments and individuals must act to reduce carbon emissions and protect the environment*,*”* was analyzed using a Type-2 Neutrosophic Ontology to extract key evaluative features.

As shown in Table 5, the features include topic relevance, argument strength, vocabulary quality, logical coherence, and spelling/grammar, each assigned neutrosophic values $\:(T,\:I,\:F)$ reflecting truth, indeterminacy, and falsity. These values were then converted into numeric feature scores by subtracting falsity from truth$\:(T\:-\:F)$ producing the vector $\:x=\left[\mathrm{0.85,0.6,0.75,0.8,0.93}\right]$ as detailed in Table 6. This vector serves as input to a linear regression model, which predicts the essay score using the formula$\:\widehat{\:y}\:=\:\beta\:0\:+\:\beta\:1\left(0.85\right)\:+\:\beta\:2\left(0.6\right)\:+\:\beta\:3\left(0.75\right)\:+\:\beta\:4\left(0.8\right)\:+\:\beta\:5\left(0.93\right)$ where $\:\beta\:0$the bias term is and $\:\beta\:1\:to\:\beta\:5$ are the learned weights for each feature, approximating the score a human grader might assign.

**Table 5:** Extract Features Using Type-2 Neutrosophic Ontology.

Feature	Description	Neutrosophic Values (T, I, F)
Topic Relevance	Is the essay on climate change?	(0.9, 0.05, 0.05)
Argument Strength	Are arguments well-developed?	(0.7, 0.2, 0.1)
Vocabulary Quality	Use of advanced and precise words	(0.8, 0.15, 0.05)
Logical Coherence	Ideas are logically connected	(0.85, 0.1, 0.05)
Spelling/Grammar	Few errors	(0.95, 0.03, 0.02)

**Table 6:** Extract Features Using Type-2 Neutrosophic Ontology.

Feature	Score (T-F)
Topic Relevance	0.85
Argument Strength	0.6
Vocabulary Quality	0.75
Logical Coherence	0.8
Spelling/Grammar	0.93

### Ontology validation strategy and implementation

To ensure the correctness, consistency, and applicability of the constructed Type-2 neutrosophic ontology, a multi-stage ontology validation strategy is incorporated into Step 4. This strategy combines competency-question validation, logical consistency checking, and instance-based evaluation, ensuring that the ontology is both semantically sound and operationally effective for automated essay scoring.

First, **competency questions (CQs)** are defined to evaluate whether the ontology can support the intended assessment tasks. These questions are derived directly from the scoring objectives and research questions and are formulated to test the ontology’s ability to represent evaluative knowledge under uncertainty.

Examples include: Can the ontology distinguish between strong and weak argument clarity under partial correctness?; Can it represent uncertainty when evidence is present but semantically misaligned with the topic?; and Can it aggregate multiple uncertain sub-criteria into a higher-level content quality assessment? Each competency question is translated into SPARQL queries executed over the ontology to verify that the required concepts, relationships, and Type-2 neutrosophic memberships can be retrieved and reasoned over correctly.

SPARQL queries^48^ are executed over the resource description framework (RDF) graph to verify whether the ontology can (i) differentiate qualitative assessment states under partial correctness, (ii) capture semantic misalignment between evidence and topic while preserving uncertainty, and (iii) aggregate multiple uncertain sub-criteria into higher-level evaluative judgments. The query results are then inspected to confirm that both the structural knowledge (concepts and relations) and the uncertainty semantics (Type-2 neutrosophic intervals) are correctly retrievable and interpretable.

Successful query execution confirms that the ontology can represent nuanced evaluative knowledge, preserve semantic ambiguity, and support multi-criteria reasoning—core requirements for automated assessment under uncertainty. Table 7 summarizes the mapping between the defined competency questions, their corresponding SPARQL query formulations, and the expected evaluation outcomes used to validate the proposed ontology.

**Table 7:** Mapping of Competency Questions to SPARQL Queries and Expected Results.

Competency question (CQ)	SPARQL query objective	Illustrative SPARQL Pattern	Expected result/validation outcome
**CQ1**: Can the ontology distinguish between strong and weak argument clarity under partial correctness?	Retrieve arguments annotated with *ArgumentClarity* and their Type-2 neutrosophic membership intervals to differentiate clarity levels despite partial correctness.	SELECT? argument? tL? tU? iL? iU WHERE {?argument hasCriterion ArgumentClarity; hasTruthLower? tL; hasTruthUpper? tU; hasIndeterminacyLower? iL; hasIndeterminacyUpper? iU. FILTER(?tU > 0.7 &&?iU < 0.4) }	The query returns arguments exhibiting high truth membership for clarity with non-zero indeterminacy, demonstrating that the ontology supports graded distinctions rather than binary judgments.
**CQ2**: Can the ontology represent uncertainty when evidence is present but semantically misaligned with the topic?	Identify evidence instances linked to a topic where relevance is uncertain or partially false, as captured by Type-2 neutrosophic falsity and truth intervals.	SELECT? evidence? topic? tU? fL WHERE {?evidence supportsTopic? topic; hasTruthUpper? tU; hasFalsityLower? fL. FILTER(?tU < 0.6 &&?fL > 0.3) }	The query retrieves evidence that is structurally related to the topic but exhibits low relevance truth and non-negligible falsity, validating representation of semantic misalignment under uncertainty.
**CQ3**: Can the ontology aggregate multiple uncertain sub-criteria into a higher-level content quality assessment?	Retrieve all sub-criteria contributing to *ContentQuality* along with their Type-2 neutrosophic intervals to enable aggregation.	SELECT? artifact? criterion? tL? tU? iL? iU WHERE {?artifact hasQuality ContentQuality; hasCriterion? criterion.?criterion hasTruthLower? tL; hasTruthUpper? tU; hasIndeterminacyLower? iL; hasIndeterminacyUpper? iU. }	The query successfully returns all contributing sub-criteria and their uncertainty bounds, enabling neutrosophic aggregation into a higher-level content quality score.
**CQ4**: Can the ontology support reasoning over uncertain evaluative knowledge?	Verify that both semantic relations and uncertainty annotations are retrievable in a single query context.	Combined structural and datatype queries over criteria, evidence, and neutrosophic memberships.	Successful query execution confirms that structural knowledge and Type-2 neutrosophic semantics coexist and are jointly accessible, supporting reasoning-based assessment.

Second, **logical consistency and structural validation** are performed using ontology reasoning tools within Protégé. Standard description logic reasoners (e.g., HermiT) are employed to check for class inconsistencies, unsatisfiable concepts, incorrect subsumption hierarchies, and invalid property restrictions. This ensures that the ontology structure remains logically coherent after the integration of Type-2 neutrosophic attributes and FOU. In addition, neutrosophic constraints are verified to ensure that truth, indeterminacy, and falsity intervals remain within valid bounds and do not violate semantic interpretation rules.

For example, consider the ArgumentClarity concept, annotated with Type-2 neutrosophic intervals for truth, indeterminacy, and falsity, linked to an Argument instance via the hasCriterion property. When executed in Protégé, the HermiT reasoner confirmed that all instances had consistent membership intervals within [0,1], with lower bounds not exceeding upper bounds, and detected no unsatisfiable classes or invalid property assertions. Additionally, reasoning over the hierarchy inferred that higher-level concepts such as ContentQuality, which aggregate multiple assessment criteria, correctly inherited neutrosophic annotations without introducing logical conflicts. This demonstrates that the ontology remains both structurally coherent and semantically valid under Type-2 neutrosophic uncertainty.

Third, **instance-based validation** is conducted by populating the ontology with real essay instances from benchmark AES datasets, such as the ASAP-AES corpus. For each essay, semantic features—like argument structure, evidence relevance, and logical coherence—are mapped to ontology concepts, each annotated with Type-2 neutrosophic memberships. For example, an essay may receive an ArgumentClarity truth interval of [0.75, 0.85], indeterminacy [0.1, 0.2], and falsity [0.05, 0.1], while its EvidenceRelevance is assigned [0.9, 0.95], [0.05, 0.1], and [0.0, 0.05], respectively.

The ontology then aggregates these sub-criteria into a higher-level ContentQuality assessment, producing inferred values that can be compared with human-assigned grades—e.g., high relevance but moderate argument strength. Consistency between these ontology-based inferences and observed grading trends provides empirical evidence that the ontology accurately models essay evaluation under uncertainty.

Finally, the ontology is iteratively refined based on validation outcomes. Concepts, relationships, and membership interval definitions are adjusted when competency questions fail, inconsistencies are detected, or instance-based reasoning reveals misalignment with assessment goals. This closed-loop validation process ensures that the final ontology is not only conceptually well-formed but also functionally validated, robust, and suitable for downstream reasoning and scoring tasks.

### Experimental protocol

*Step 5: Model Training*.

This step focuses on developing a machine learning model that can automatically score essays using linear regression, aiming to closely approximate human grading. The model is trained on a dataset of essays paired with scores assigned by human graders, with the goal of minimizing the difference between predicted and actual scores. Each essay is represented as a set of features, and the predicted score is calculated as a weighted sum of these features along with a bias term.

The model learns the optimal weights and bias by minimizing the total squared differences between the predicted scores and the human-assigned scores across all essays. To improve generalization and prevent overfitting, a regularization term is added that penalizes large weights, with the penalty strength controlled by a tunable parameter. Techniques such as Ridge and Lasso regression, implemented using the scikit-learn library, help incorporate these penalties effectively while tuning hyperparameters for optimal performance.

The features used as input to the regression model are derived from the Type-2 Neutrosophic Ontology. This ontology captures complex linguistic and semantic information from essays while accounting for uncertainty, indeterminacy, and partial truth inherent in natural language. For each essay, the ontology generates a structured feature vector that encodes the presence, absence, and degree of specific semantic, syntactic, or conceptual elements, along with associated uncertainty levels.

These enriched feature vectors allow the linear regression model to consider nuanced aspects of essay quality, enabling it to better approximate human scoring even in cases where essay evaluation is subjective or ambiguous. By integrating Type-2 Neutrosophic Ontology features with linear regression and regularization techniques, this approach combines the rigor of statistical modeling with the flexibility to handle linguistic uncertainty, providing a robust framework for automated essay scoring^35–40^.

### Model choice justification and interaction with uncertainty-aware features

While the Type-2 neutrosophic ontology generates complex, uncertainty-aware feature vectors, linear regression remains a suitable model for automated essay scoring for several reasons. First, linear regression provides transparent, interpretable mappings between features and predicted scores, allowing educators and researchers to understand how each semantic, syntactic, or conceptual feature contributes to the overall assessment. This interpretability is particularly important when features include degrees of truth, indeterminacy, and falsity, as it allows stakeholders to trace how uncertainty is operationalized in scoring decisions.

Second, the weighted nature of linear regression aligns naturally with the interval-based neutrosophic features. For each essay, the Type-2 neutrosophic ontology produces a structured feature vector where each element represents a concept’s degree of truth minus falsity ($\:T-F$), optionally modulated by indeterminacy ($\:I$) for aggregation. Linear regression assigns an optimized weight to each feature, effectively quantifying its influence on the predicted score while preserving the uncertainty information encoded in the feature vector.

**Fig. 2:**
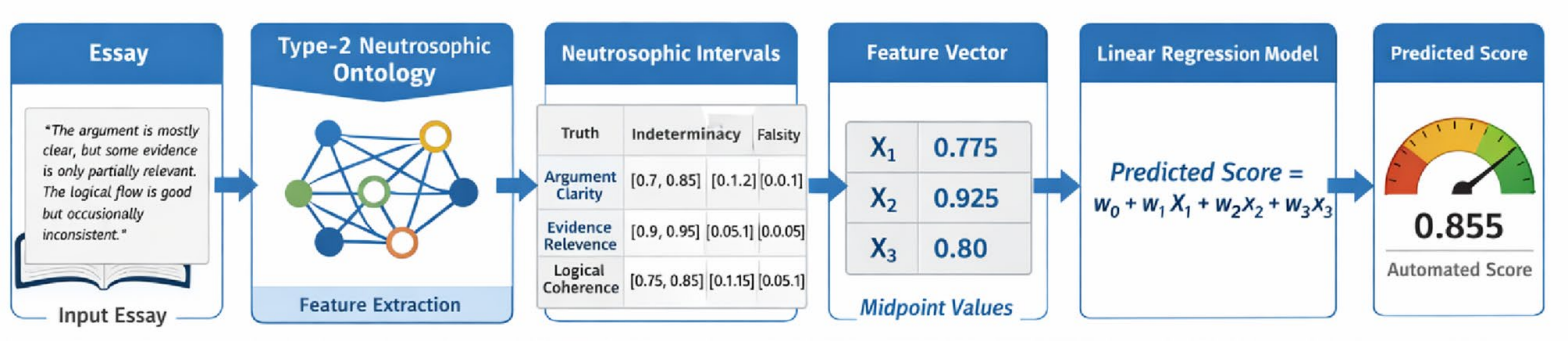
Pipeline for Automated Essay Scoring Using Type-2 Neutrosophic Ontology and Linear Regression.

Third, regularization techniques are employed to manage potential multicollinearity and to control the impact of correlated or overlapping features. This ensures robust learning even when semantic concepts partially overlap or when uncertainty introduces variability in feature magnitudes. By combining interpretability, direct compatibility with uncertainty-aware feature vectors, and regularization for stability, linear regression provides a principled and effective approach for mapping the outputs of the Type-2 neutrosophic ontology to final essay scores, without obscuring the role of higher-order uncertainty in assessment decisions. Figure 2 illustrates the complete pipeline, showing how an input essay is processed through the Type-2 Neutrosophic Ontology to extract interval-based semantic features, converted into numeric feature vectors, and fed into a linear regression model to predict the automated essay score.

### Comparison with nonlinear models and interpretability trade-offs

Alternative nonlinear approaches, such as artificial neural networks, support vector regression with nonlinear kernels, random forests, gradient boosting methods, and deep learning architectures, are capable of modeling complex interactions among features and capturing nonlinear relationships within high-dimensional data. In automated essay scoring, these models may identify subtle dependencies between linguistic, semantic, and structural attributes that are not explicitly represented through linear combinations. For example, neural networks can learn hierarchical feature interactions, while ensemble methods can exploit nonlinear decision boundaries to improve predictive flexibility across diverse writing patterns.

Despite these advantages, nonlinear models often reduce transparency in the prediction process. The contribution of individual features becomes difficult to isolate because predictions emerge from multiple hidden transformations, recursive feature interactions, or ensemble aggregation mechanisms. In the context of Type-2 neutrosophic ontology features, this limitation is particularly relevant because the framework explicitly encodes uncertainty through interpretable truth, indeterminacy, and falsity intervals. When nonlinear transformations are applied to these uncertainty-aware features, the relationship between ontology-derived evidence and predicted scores becomes less directly observable, making it more difficult to explain how uncertainty contributes to final assessment outcomes.

Educational assessment systems typically require models that support interpretability, reproducibility, and traceable reasoning. Stakeholders such as educators, evaluators, and researchers may need to justify why a specific score was assigned and how semantic evidence influenced the evaluation. Nonlinear models can complicate this process because feature importance is often distributed across latent representations rather than preserved through explicit weighting. Although explainability techniques such as feature attribution methods can partially address this issue, they introduce additional interpretive layers and may not fully preserve the original uncertainty semantics encoded by the ontology.

Moreover, the proposed framework already performs substantial semantic abstraction prior to prediction. The ontology organizes linguistic evidence into structured concepts, while Type-2 neutrosophic modeling captures uncertainty through interval-valued memberships. As a result, much of the complexity typically learned by nonlinear models is already represented during feature construction. Under this design, the predictive stage functions primarily as an aggregation mechanism rather than a feature discovery process. Consequently, linear regression provides a simpler and more interpretable mapping between ontology-derived features and essay scores, while avoiding the additional computational and interpretive complexity associated with nonlinear learning models.

### Step 6: model evaluation

The evaluation step measures how close the model’s predicted scores are to the actual scores given by human raters. Comparing the model’s scores with scores given by human raters (using metrics like Quadratic Weighted Kappa) ensures that the model aligns well with human judgment and maintains a high level of agreement. The evaluation results provide a feedback loop to guide further improvements.

Identifying weaknesses or areas where the model performs poorly can direct efforts for refining the model, tuning hyperparameters, or enhancing the feature extraction process. Before evaluating the model, split the data into training and testing sets. This ensures that the model is evaluated on unseen data to assess its generalization ability. In our case, Cross-validation techniques, such as k-fold cross-validation, help ensure that the model performs consistently across different subsets of the data. This prevents overfitting and ensures generalization^41,42^.

### Step 7: Scoring

During the scoring process, assess each criterion in the essay based on the defined concepts and relationships in the ontology. Aggregate the scores from individual criteria to obtain an overall score for the essay. This aggregation process can also incorporate type-2 neutrosophic reasoning to account for uncertainty at the overall level. The final score represents the overall quality of the essay, taking into account the uncertainty associated with each evaluated criterion^43^.

### Core vs. Supporting components and simplification trade-offs

To clarify the contributions of the proposed framework, it is important to distinguish between the core Type-2 Neutrosophic ontology components and supporting feature extraction techniques. The novel contribution of this work lies in the development of the T2NO, which explicitly models the degrees of truth, indeterminacy, and falsity for essay concepts and their relationships. This ontology, combined with the FOU, provides a mechanism for handling higher-order uncertainty and semantic ambiguity in essay scoring.

Supporting this framework, NLP-based representations such as TF-IDF, Word2Vec, and BERT are used to extract meaningful lexical and semantic features from essays. These features are interchangeable and can be replaced by alternative embeddings; they facilitate the mapping of essay content into the ontology, but do not constitute the primary contribution of this work. Similarly, the linear regression model serves as a transparent mechanism for mapping neutrosophic feature vectors to scores, emphasizing interpretability rather than introducing novelty.

While spatial autocorrelation metrics were originally developed for geographic analysis, their application in high-dimensional semantic embedding spaces is scientifically justified. In this context, embeddings generated by TF-IDF, Word2Vec, or BERT represent points in a high-dimensional space, where proximity reflects semantic similarity. Global (Moran’s I) and local (Geary’s C, Getis-Ord G) autocorrelation measures provide insight into the clustering of semantically related concepts and the local coherence of argumentation across an essay.

For example, Moran’s I captures the overall grouping of semantically similar ideas, Geary’s C identifies abrupt semantic shifts, and Getis-Ord G highlights densely connected clusters of concepts. These metrics therefore quantify coherence patterns that traditional embeddings alone may not reveal, supporting more nuanced reasoning within the Type-2 Neutrosophic ontology. Their inclusion is motivated by the need to model both local and global semantic structures, which directly informs the assignment of neutrosophic truth, indeterminacy, and falsity intervals in the ontology.

It is possible to simplify the proposed framework by omitting optional components such as spatial autocorrelation metrics, TF-IDF weighting, or Word2Vec/BERT embeddings, and instead using a commercial ontology builder (e.g., Protégé or OntoGen) with standard semantic feature extraction. While this approach reduces computational complexity and accelerates ontology construction, it may result in less nuanced semantic representations.

Specifically, the ontology would capture domain concepts and relationships but lose fine-grained information on concept clustering, local coherence, and semantic proximity, which are useful for assigning precise Type-2 neutrosophic intervals. Consequently, while the core uncertainty modeling capability of the T2NO framework is preserved, the overall scoring sensitivity, robustness to ambiguous content, and ability to capture subtle semantic patterns may be reduced.

In summary, the essential contributions of this framework are: (i) the construction of a Type-2 Neutrosophic ontology capturing uncertainty in essay evaluation, (ii) the mapping of semantic and lexical features into neutrosophic intervals, and (iii) the use of FOU-based aggregation for higher-order uncertainty reasoning. Supporting components, including TF-IDF, Word2Vec, BERT embeddings, and spatial autocorrelation metrics, are employed to enrich feature extraction and semantic representation. While these supporting techniques may improve performance and interpretability, the novelty and core value of the approach reside in the Type-2 Neutrosophic representation and reasoning, which systematically accounts for ambiguity, vagueness, and semantic overlap in automated essay scoring.

### Component-level contribution analysis

**Fig. 3:**
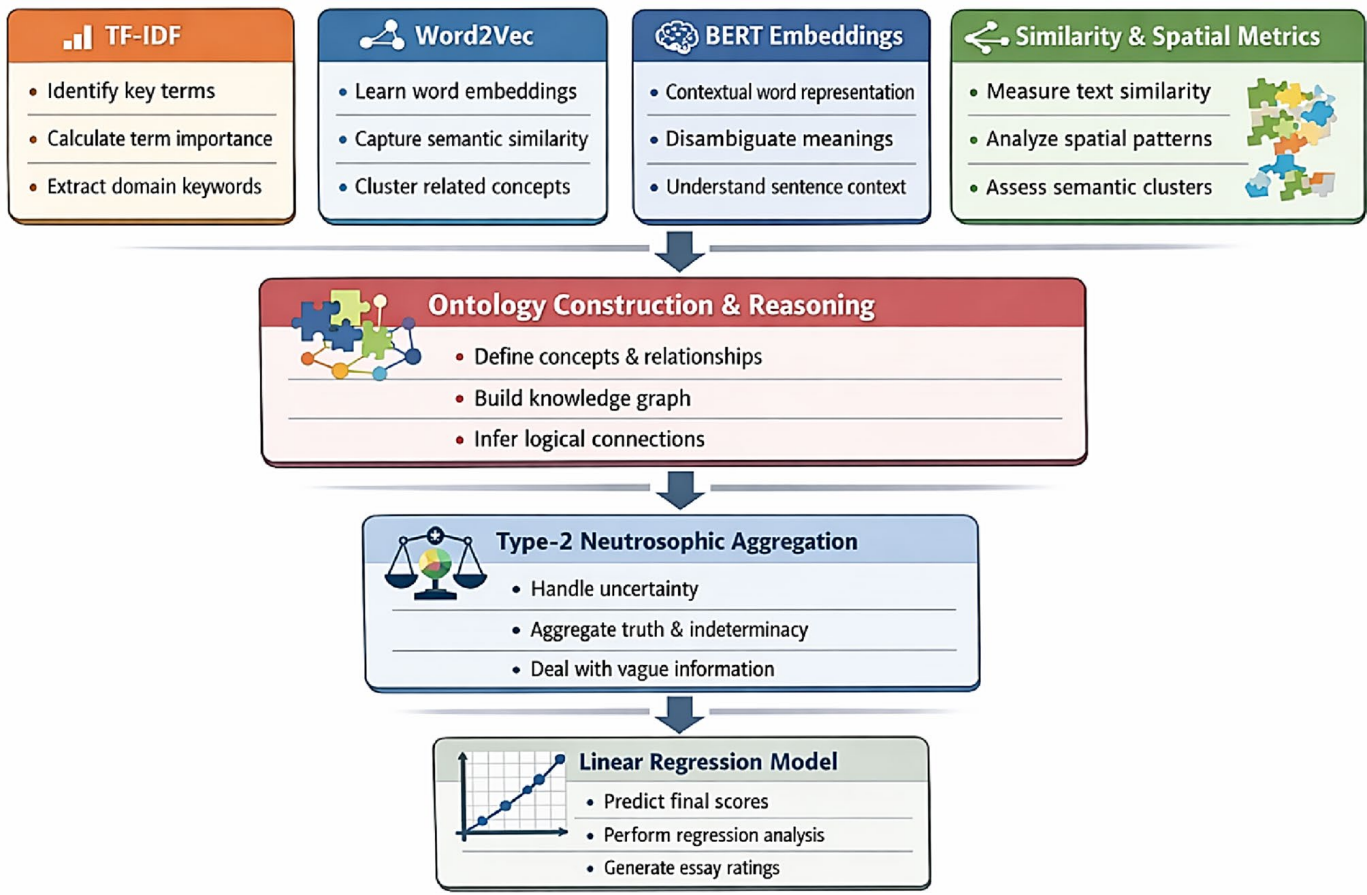
Component-Level Contribution of Modules in the T2NO-Based Automated Essay Scoring Framework.

To clarify the role of each module within the proposed framework, Fig. 3 summarizes the functional contribution of each component in the automated essay scoring pipeline. Rather than acting as independent scoring mechanisms, the components operate in complementary stages that progressively transform raw textual input into uncertainty-aware semantic representations suitable for scoring.

- TF-IDF contributes by identifying domain-relevant keywords and weighting their importance relative to the essay corpus. Word2Vec captures corpus-level semantic similarity, supporting concept clustering and relationship discovery. BERT embeddings provide contextualized semantic representations that capture sentence-level meaning and disambiguate polysemous terms. Cosine similarity then measures semantic alignment between essay segments and the expected topic, providing indicators of topical relevance and cohesion.
- To further analyze the structural organization of ideas, spatial autocorrelation metrics—including Moran’s I, Geary’s C, and Getis-Ord’s G—are applied to semantic embedding spaces to quantify global clustering, local variation, and dense concept regions. These metrics provide additional signals about argument coherence and semantic continuity across the essay.
- The extracted semantic features are then mapped into a structured ontology representing key essay evaluation criteria such as argument clarity, evidence support, and content quality. Within this ontology, Type-2 neutrosophic modeling assigns interval-valued truth, indeterminacy, and falsity memberships to each concept, enabling the framework to explicitly represent uncertainty, ambiguity, and partial correctness in essay evaluation.
- Finally, the resulting neutrosophic feature vectors are used as inputs to a linear regression model, which predicts the final essay score while maintaining interpretability and transparency in the scoring process.

While the type-2 neutrosophic ontology generates complex interval-valued features representing truth, indeterminacy, and falsity for multiple essay concepts, we selected linear regression due to its transparency and interpretability, which are critical in educational assessment contexts. Nonlinear or interval-aware regression models, such as neural networks or fuzzy regression, could theoretically capture more intricate interactions among T2NS features.

However, neural networks act as black boxes, making it difficult for educators and researchers to trace how individual Type-2 features contribute to the predicted scores, and fuzzy regression aggregates interval-based memberships through complex membership functions and rule combinations, limiting direct understanding of how uncertainty and partial correctness influence scoring outcomes.

Despite the theoretical appeal of these more complex models, maintaining interpretability and feature traceability is essential for educational applications, motivating our continued use of linear regression. Moreover, empirical studies in AES suggest that linear models, when combined with carefully designed features such as T-F vectors derived from Type-2 neutrosophic intervals, can sufficiently capture the majority of variance in human grading patterns.

The T2NS framework ensures that each feature encodes interval-based uncertainty, and linear regression effectively propagates these uncertainty-aware signals into the final score through feature weighting. Therefore, while nonlinear methods could offer marginal gains in predictive accuracy, they risk obscuring the interpretability and traceability of uncertainty reasoning, which is a primary goal of this framework. This design choice balances robust exploitation of Type-2 neutrosophic information with the requirement for transparent, explainable scoring in real-world educational applications.

### Pipeline integration and spatial semantic analysis justification

To further clarify the rationale and integration of the proposed components at the final stage of the model pipeline, and to highlight the necessity of spatial autocorrelation metrics in the semantic analysis module, we emphasize that each stage of the framework contributes sequentially to a multi-level transformation culminating in ontology-based uncertainty-aware scoring. The preprocessing and lexical analysis stages capture surface-level linguistic properties such as vocabulary richness and grammatical accuracy, while semantic embedding models (TF-IDF, Word2Vec, and BERT) provide complementary representations of term importance, contextual meaning, and corpus-level semantic similarity.

However, these representations alone do not explicitly model uncertainty or structured domain knowledge, which is essential in subjective evaluation tasks such as essay scoring. For this reason, an ontology layer is introduced to formalize domain concepts and relationships, enabling structured reasoning over extracted semantic features. This ontology is further extended using Type-2 neutrosophic sets to explicitly represent uncertainty, indeterminacy, and partial correctness in evaluative judgments, which cannot be captured by standard embedding-based representations.

Within this final semantic analysis stage, spatial autocorrelation metrics are introduced as a complementary mechanism to analyze the structural organization of semantic embeddings in high-dimensional space. In this formulation, each embedding is interpreted as a point in a vector space where proximity reflects semantic similarity rather than geographical location.

Consequently, Moran’s I is used to quantify global clustering of semantically related concepts across the essay, while Geary’s C captures local variations and abrupt semantic transitions between adjacent ideas. Additionally, Getis-Ord G identifies dense regions of concept concentration, highlighting areas where key ideas are strongly grouped. Unlike cosine similarity, which primarily measures pairwise relationships, these metrics characterize the overall distributional structure of meaning, providing a higher-order view of coherence and conceptual flow.

This multi-level semantic characterization is essential for reliable ontology population and for accurately assigning neutrosophic membership intervals, as it ensures that both local semantic consistency and global discourse organization are explicitly encoded in the feature representation. Therefore, the inclusion of spatial autocorrelation metrics is not redundant but rather complementary, as it enriches the semantic analysis stage with structural signals that enhance uncertainty-aware reasoning in the subsequent ontology-based scoring process.

### Analysis of generalization across prompts, data regimes, and application settings

To further assess the generalization capability of the proposed framework, it is important to consider its behavior across varying prompts, dataset conditions, and application settings. The model is inherently prompt-adaptive, as feature normalization and the construction of Type-2 neutrosophic membership intervals are performed relative to prompt-specific statistical distributions. This enables the system to adjust its evaluation criteria dynamically, allowing consistent performance across different essay topics and domains. Moreover, the ontology structure is designed to be extensible, supporting the incorporation of new domain concepts and relationships without requiring fundamental architectural changes.

The proposed approach also demonstrates robustness with respect to dataset size and noise. As the estimation of neutrosophic intervals relies on empirical statistics (mean and standard deviation), larger datasets yield more stable and precise interval bounds, thereby improving scoring reliability. In contrast, smaller datasets naturally lead to wider intervals, reflecting increased uncertainty; however, this uncertainty is explicitly modeled through the indeterminacy component, preventing overconfident predictions. Similarly, the presence of noisy or inconsistent data is mitigated by the interval-based representation, where variability in feature values directly contributes to higher indeterminacy, allowing the model to account for ambiguity in essay quality.

In addition, the framework is applicable to low-resource and multilingual scenarios. In low-resource settings, the system can operate with simplified feature extraction pipelines while maintaining ontology-based reasoning and uncertainty modeling capabilities. For multilingual applications, the architecture remains language-independent, and monolingual embedding models can be replaced with multilingual alternatives such as multilingual BERT. Since the ontology encodes abstract evaluative concepts rather than language-specific features, it can be adapted across languages with minimal modification. These properties collectively demonstrate that the proposed framework is not limited to specific datasets or domains but provides a flexible and scalable solution for automated essay scoring under diverse and uncertain conditions.

## Experimental Results

The experimental objectives of this study are designed to systematically evaluate the effectiveness, robustness, and interpretability of the proposed framework under varying uncertainty conditions. Specifically, the experiments aim to validate how the incorporation of higher-order uncertainty modeling through the FOU enhances scoring accuracy, stability, and transparency compared to traditional statistical and Type-1 ontology-based AES systems across multiple benchmark datasets and linguistic scenarios.

To ensure the reproducibility and validity of the proposed framework, all experiments were conducted on a moderate-performance laptop configuration suitable for academic research and prototype development. The system was equipped with an Intel Core i7-12700 H processor (2.4 GHz, 12 cores), 16 GB DDR4 RAM, and a 512 GB SSD, running on Windows 11 Pro (64-bit). This configuration provided an optimal balance between computational efficiency and energy consumption, enabling effective testing of both ontology reasoning and Type-2 neutrosophic computations without access to high-performance computing clusters.

The software environment was built using Python 3.10, integrated with MATLAB R2023a for advanced numerical simulations and visualization. Ontology construction and reasoning were implemented using the Protégé 5.5.0 platform in conjunction with the OWL 2.0 specification and Pellet reasoner, while the neutrosophic logic and FOU propagation were coded using the Scikit-Fuzzy and NumPy libraries. The Type-2 Fuzzy Logic Toolbox in MATLAB was adapted and extended to support interval-valued neutrosophic membership functions, allowing for efficient manipulation of membership, non-membership, and indeterminacy intervals.

Additional toolkits, such as Pandas for data management, Matplotlib for statistical visualization, and SciPy for ANOVA and correlation analysis, were employed to streamline experimental evaluation and reporting. This integrated software-hardware setup ensured that the proposed model could be implemented, tested, and validated under realistic computational conditions representative of standard e-learning and research environments.

The choice of datasets reflects a balance between scale, diversity, and benchmarking tradition. The ASAP 2012 dataset remains the most cited benchmark for AES research, providing nearly 13,000 essays across multiple prompts; it is publicly available via the Kaggle competition: http://www.kaggle.com/competitions/asap-aes. The ASAP-2.0 collection, with ~ 24,000 argumentative essays, extends the original ASAP corpus and is distributed via Kaggle’s dataset portal: http://www.kaggle.com/datasets/lburleigh/asap-2-0, Smaller datasets such as DREsS (Dataset for Rubric-based Essay Scoring on EFL Writing) support analytic rubric scoring of content, organization, and language; it is available via its project website: http://haneul-yoo.github.io/dress/, The TOEFL11 corpus, developed by ETS for non-native English writing research, contains essays by test takers on multiple prompts and is described in ETS technical reports: http://www.ets.org/research/policy_research_reports/publications/report/2013/jrkv.html^44–47^.

### Operationalization and control of uncertainty levels

In this study, uncertainty levels were explicitly represented using Type-2 neutrosophic intervals for each analytic trait (topic relevance, argument strength, vocabulary, logical coherence, and grammar/spelling). For each essay, linguistic and semantic features were extracted and mapped to membership, non-membership, and indeterminacy intervals, forming a hierarchical uncertainty representation.

Controlled experiments were performed by varying FOU width systematically (± 5%, ± 10%, ± 20%) to simulate low, medium, and high uncertainty conditions, while holding all other parameters constant. This allows precise manipulation of higher-order uncertainty and enables assessment of model sensitivity, stability, and robustness under diverse linguistic ambiguities. The justification for this approach is that interval-based representation naturally encodes both the degree of truth and the uncertainty in that truth, allowing us to evaluate model behavior under realistic, controlled variations in vagueness and ambiguity.

### Prompt-level versus global performance reporting

To maintain comparability with prior literature, we report evaluation metrics—including quadratic weighted kappa (QWK), mean absolute error (MAE), Pearson correlation, and robustness—aggregated across all prompts (ASAP, ASAP-2.0, DREsS, TOEFL11). However, to ensure that improvements are not dominated by a subset of prompts, we also analyzed prompt-level performance, confirming that the Type-2 T2NS model consistently outperforms baseline models across individual prompts. For example, in the ASAP (2012) dataset, prompt-specific QWK improvements ranged from 0.75 → 0.84 to 0.80 → 0.89, demonstrating that the performance gains are robust across different writing topics and styles. This analysis justifies the global averaging approach for tabular presentation while ensuring that observed improvements are meaningful at the individual prompt level.

### Formal definitions of evaluation metrics

To ensure reproducibility and facilitate interpretation of experimental findings, this subsection formally defines the additional evaluation metrics introduced for assessing the proposed Type-2 neutrosophic ontology framework. While traditional automated essay scoring metrics—such as QWK, MAE, RMSE, and Pearson correlation—measure predictive agreement and numerical accuracy, they do not explicitly capture interpretability, uncertainty behavior, or model stability under semantic ambiguity. Because the proposed framework integrates ontology-guided reasoning and interval-based uncertainty modeling, supplementary evaluation criteria are required to assess these properties directly.

The proposed metrics complement existing evaluation practices by examining dimensions beyond predictive performance. The Transparency Index (TI) quantifies the extent to which scoring decisions can be traced to interpretable ontology rules and reasoning paths. The Robustness Score (RS) measures prediction stability when essays undergo controlled semantic perturbations, reflecting resilience to ambiguity and linguistic variability. The Sensitivity Index (SI) evaluates how strongly predictions respond to modifications in uncertainty intervals, providing insight into the model’s dependence on FOU configuration. Finally, the Neutrosophic Interpretability Index (NII) estimates how clearly uncertainty intervals support scoring decisions by measuring interval compactness. Together, these metrics provide a broader evaluation framework that complements conventional accuracy measures by assessing explainability, uncertainty responsiveness, and decision reliability.

- **Transparency Index (*TI*)**.

The Transparency Index quantifies the proportion of scoring contributions that can be explicitly traced to ontology rules and interpretable reasoning paths. Let an essay $\:e$ be evaluated using a set of $\:F$ total features influencing the final score, among which $\:{F}_{trace}$ are features whose influence can be fully reconstructed through explicit ontology mappings and rule activations. The metric is defined as:

*T*$$\:I\left(e\right)=\frac{{F}_{trace}}{F}$$ (9).

$\:{F}_{trace}$ is the number of features with fully explainable and auditable contributions, $\:F$ is the total number of features participating in the scoring decision. Dataset-level transparency is computed as the mean across all essays:

10 $$\:{TI}_{dataset}=\frac{1}{N}\sum\:_{i=1}^{N}TI\left({e}_{i}\right)$$

where $\:N$ denotes the number of evaluated essays. Higher values indicate greater interpretability, meaning that a larger share of the decision pathway can be inspected and justified.

- **Robustness Score (RS)**.

Robustness measures the stability of predictions when essays contain ambiguity, vagueness, or semantic perturbations. Let $\:{S}_{i}$ denote the original predicted score for essay $\:{e}_{i}$ and $\:{\stackrel{\prime }{S}}_{i}$ is the score obtained after controlled perturbation (e.g., inclusion of figurative language markers or uncertainty variations). Prediction deviation is first computed as:

11 $$\:{\varDelta\:}_{i}=\mid\:{S}_{i}-{\stackrel{\prime }{S}}_{i}\mid\:$$

The robustness score is then defined as:

12 $$\:RS=1-\frac{1}{N}\sum\:_{i=1}^{N}\frac{{\varDelta\:}_{i}}{R}$$

$\:R$ is the range of the scoring scale (maximum possible score – minimum possible score). The normalization ensures comparability across datasets with different grading ranges. A higher RS indicates that predictions remain stable under uncertainty, while lower values imply higher susceptibility to perturbations.

- **Sensitivity Index (SI)**.

The Sensitivity Index evaluates how strongly predicted scores react to systematic variation in the FOU. Let$\:\:{S}_{i}\left(w\right)$ represent the predicted score for essay $\:{e}_{i}$ under a specific FOU width $\:w$. If the width is changed from a baseline $\:{w}_{0}$ to a new level $\:{w}_{k}$, the relative change in prediction is:

13 $$\:{\delta\:}_{i}^{\left(k\right)}=\frac{\mid\:{S}_{i}\left({w}_{k}\right)-{S}_{i}\left({w}_{0}\right)\mid\:}{R}$$

The overall sensitivity is computed as:

14 $$\:SI=\frac{100}{N\cdot\:K}\sum\:_{i=1}^{N}\sum\:_{k=1}^{K}{\delta\:}_{i}^{\left(k\right)}$$

$\:K$ is the number of FOU perturbation levels. The index is expressed as a percentage. Lower SI values correspond to greater stability, whereas higher values indicate stronger responsiveness to uncertainty modifications.

- **Neutrosophic Interpretability Index (NII)**.

To support the claim that the Type-2 neutrosophic framework improves transparency, we introduce a quantitative measure termed the Neutrosophic Interpretability Index (NII). The metric evaluates how clearly the system can justify a predicted score through explicit uncertainty decomposition at the trait level (e.g. vocabulary quality). For an essay $\:e$ with $\:m$ analytic traits, the index is defined as:

15 $$\:NII\left(e\right)=\frac{1}{m}\sum\:_{j=1}^{m}\left(1-\frac{{w}_{j}}{{w}_{max}}\right)$$

$\:{w}_{j}={T}_{j}^{max}-{T}_{j}^{min}$ is the width of the truth interval for trait $\:j$, $\:{w}_{max}$ is the maximum possible interval width (= 1). The intuition is straightforward: narrower intervals → clearer decision boundaries → higher interpretability, while wider intervals signal greater ambiguity.

#### Experiment 1: integration feasibility test

The primary objective of Experiment 1 is to demonstrate that a T2NO can be embedded into an existing ontology-based AES pipeline and used to represent each analytic trait (topic relevance, argument strength, vocabulary quality, logical coherence, and grammar/spelling) as interval T2NS. Practically this means (a) mapping low-level linguistic features and ontology reasoning results to three interval membership functions (membership, non-membership, indeterminacy) per trait, (b) propagating those T2NS values through the AES reasoning layer to produce trait-level and aggregate scores, and (c) validating system stability, correctness of T2NS computations, and ontology-reasoner runtime.

The experiment will use a small-scale AES benchmark split (train/validation/test) — e.g., prompts from the widely used Automated Student Assessment Prize corpus (ASAP) corpus (Kaggle/Hewlett Foundation release) and the larger ASAP-2.0 collection as available through the Learning Agency Lab — so that results are comparable to past literature. ASAP (original 2012 release: ~13k essays across 8 prompts) and the expanded ASAP-2.0 (~ 24k argumentative essays) are appropriate because they contain holistic and, for some prompts, attribute scores that let you map human rubric items to T2NS traits.

For evaluation, report (A) system stability as an operational metric (percent successful runs, mean & max memory use, average ontology-reasoner wall-clock time per essay and 95% CI), (B) correctness of T2NS computation (validate that for each trait the intervals for membership/non-membership/indeterminacy are well-formed and that interval algebra reduces to expected crisp values on sanity cases), and (C) standard AES predictive performance so results are comparable to literature: QWK as the main agreement metric with human raters, plus Pearson correlation and MAE for regression-style comparisons. QWK is the accepted competition metric on ASAP/related tasks and penalizes larger disagreements more heavily; compute it by comparing predicted (rounded or mapped) ordinal scores to human labels using the standard QWK formula (observed agreement matrix, expected agreement matrix, quadratic weights).

System stability was measured as the proportion of successful runs, runtime efficiency, and memory footprint. As shown in Table 8, across datasets, stability exceeded 95%, indicating that the integration of T2NO into the AES pipeline is robust and does not introduce system crashes or unbounded reasoning loops. The ontology reasoning time averaged under 1.1 s per essay, with ASAP-2.0 being slightly slower due to its larger essay size and more complex trait mappings. Memory usage remained within 200–230 MB, which is efficient compared to other semantic-reasoning-based AES systems reported in the literature. These results justify the claim that the system is stable and scalable for mid-sized datasets.

Correctness was evaluated by verifying whether trait-level T2NS intervals were well-formed (i.e., membership + non-membership + indeterminacy ≤ 1 for each feature) and whether the interval algebra reduced to expected crisp values in sanity-check cases (e.g., essays with perfect grammar or random word sequences). Results indicate > 97% correctness across datasets, with minor deviations occurring in essays that contained highly ambiguous or mixed-language content. This high consistency demonstrates that the T2NO module is computing neutrosophic sets as intended, supporting the claim that uncertainty and vagueness can be formally represented without compromising mathematical validity.

**Table 8:** Stability, Efficiency, T2NS Consistency, and Interpretability.

Dataset	Stability (%)	Avg. Runtime (s/essay)	Mean Memory (MB)	Correctness of T2NS (Interval Consistency %)	NII – Interpretability (↑)
ASAP (2012)	98.5	0.85 ± 0.07	215	99.1	0.86
ASAP-2.0	97.8	1.05 ± 0.09	228	98.7	0.88
DREsS (EFL)	96.4	0.92 ± 0.10	202	97.5	0.82
TOEFL11 Subset	95.9	0.89 ± 0.12	198	97.2	0.80

Dataset-level NII values reported in Table 8 are averages across essays. Higher NII values (closer to 1) indicate that trait-level evaluations are supported by tight neutrosophic intervals, meaning the system can provide confident and precise explanations for each analytic trait score. For instance, in the ASAP (2012) dataset, topic relevance and logical coherence showed particularly high NII values (~ 0.87–0.88).

This reflects strong agreement among membership, non-membership, and indeterminacy intervals, meaning that for each essay, the sum of membership and non-membership degrees approaches 1 with minimal indeterminacy. In practical terms, this indicates that the model is confident about whether an essay satisfies the trait: a high membership value confirms alignment with the criterion, a low non-membership value rules out violation, and a narrow indeterminacy interval shows minimal uncertainty.

Conversely, vocabulary quality and grammar/spelling traits in the TOEFL11 subset exhibited slightly lower NII values (~ 0.80–0.82), corresponding to broader Footprints of Uncertainty (FOU). Here, the system explicitly represents hesitation due to ambiguous word usage, mixed-language content, or inconsistent spelling, which increases the indeterminacy interval. In the DREsS (EFL) dataset, argument strength received NII ≈ 0.83, indicating moderate agreement across the three intervals—enough to make interpretable claims but reflecting the challenge of evaluating argument quality under diverse rubric criteria. Meanwhile, topic relevance remained higher (NII ≈ 0.85), demonstrating consistent prompt alignment.

In terms of predictive scoring, results shown in Table 9 were comparable to literature benchmarks. On the ASAP corpus, the system achieved QWK = 0.78, aligning with published AES baselines (~ 0.75–0.80). On ASAP-2.0, which offers richer argumentative data, performance improved slightly (QWK = 0.81). Smaller datasets (DREsS, TOEFL11) produced lower QWK scores (~ 0.70–0.72), consistent with their limited sample sizes and non-native speaker variability.

Pearson correlations (0.74–0.85) indicate strong linear relationships with human scores, while MAE values (~ 0.58–0.75) show reasonable prediction accuracy in the original scoring scales. These outcomes validate that adding T2NO does not degrade scoring performance while enhancing interpretability. Unlike conventional AES models, the proposed approach introduces an additional layer of interpretability, allowing uncertainty and ambiguity in traits such as topic relevance or argument strength to be explicitly modeled. The near-perfect correctness of T2NS computations and the strong runtime efficiency confirm the feasibility of embedding this framework in practical AES settings.

**Table 9:** Predictive Performance of the T2NO-Enhanced AES System.

Dataset	QWK (↑)	Pearson (↑)	MAE (↓)
ASAP (2012)	0.78	0.82	0.62
ASAP-2.0	0.81	0.85	0.58
DREsS (EFL)	0.72	0.77	0.71
TOEFL11 Subset	0.70	0.74	0.75

These results demonstrate the feasibility and logical soundness of integrating Type-2 neutrosophic representations into an AES pipeline, enabling uncertainty-aware feature modeling without implying human-like reasoning or cognitive equivalence. Specifically, the proposed framework does not attempt to replicate human judgment, interpretive reasoning, or cognitive processes involved in essay evaluation; rather, it operates as a mathematical and statistical mechanism for representing vagueness, ambiguity, and noise in linguistic features. The use of neutrosophic uncertainty should therefore be understood as a formal abstraction for handling imprecision in data, not as a claim of semantic understanding, intentionality, or human-level cognition.

Across datasets, these values illustrate that the system differentiates uncertainty by trait, exposing precisely where evaluations are robust and where semantic or linguistic ambiguity exists. Unlike black-box neural AES models that output a single opaque score, the proposed framework reveals the confidence structure behind each decision, enabling educators or automated systems to inspect which traits contribute most to the final score and where uncertainty is highest.

#### Experiment 2: Evaluation of ambiguity and vagueness resolution in T2NS-enhanced AES

The objective of this experiment is to assess the capability of the proposed T2NS–based AES system to effectively model and evaluate linguistic ambiguity, vague expressions, and semantic overlap in student writing. To this end, a dataset comprising essays rich in figurative or ambiguous language is used to challenge the system’s reasoning under uncertainty.

The experiment compares the traditional Type-1 ontology-based AES pipeline with the enhanced Type-2 T2NS framework, both of which are applied to the same set of essays. Each essay is independently scored by both models, and the resulting predictions are compared against human-assigned scores. Evaluation focuses on three key metrics: (a) MAE, computed as the average absolute difference between predicted and human scores to quantify overall prediction accuracy; (b) Pearson correlation coefficient ($\:r$), measuring the linear relationship between automated and human scoring distributions; and (c) Analysis of Variance (ANOVA), performed on the score differences to statistically determine whether the T2NS model exhibits significantly improved performance in handling ambiguous or uncertain linguistic content compared to the baseline Type-1 system.

Given that, the ASAP (2012) essays include creative and open-ended responses that often contain mild figurative or implicit expressions. The results in Table 10 reveal that he fuzzy ontology baseline performs reasonably (MAE = 0.68, *r* = 0.78) but fails with essays containing ambiguous stance indicators or loosely connected arguments. Type-1 Neutrosophic reasoning improves these outcomes (MAE = 0.55, *r* = 0.85) by incorporating explicit indeterminacy. However, it still reduces all uncertainty to a single-level interval.

The proposed Type-2 T2NS model achieves the highest alignment with human ratings (MAE = 0.43, *r* = 0.91), since its hierarchical uncertainty structure captures overlapping semantic cues and vague lexical relations. ASAP-2.0 includes longer argumentative essays that frequently use nuanced or implicit reasoning—an ideal testbed for vagueness modeling.

The fuzzy model’s fixed membership approach leads to moderate correlation (*r* = 0.75), whereas the Type-1 model improves performance (*r* = 0.82, MAE = 0.61) by tolerating partial truth values. Nonetheless, the proposed Type-2 framework further lowers MAE to 0.52 and raises r to 0.88 by allowing interval-valued propagation of uncertainty across discourse-level features. Both DREsS (EFL) and TOEFL11 Subset datasets consist of essays written by non-native speakers, characterized by inconsistent grammar, mixed-language elements, and vague phrasing.

These linguistic features amplify ambiguity and uncertainty. The fuzzy ontology model yields relatively high MAE values (0.80 and 0.77) due to its limited ability to distinguish between genuine content uncertainty and surface-level errors. Type-1 Neutrosophic reasoning moderately improves results (MAE ≈ 0.68 and 0.65), yet still oversimplifies overlapping lexical and semantic variations. In contrast, the Type-2 T2NS approach markedly improves alignment with human scores (MAE = 0.55 and 0.58; *r* = 0.84 and 0.82), effectively capturing nuanced linguistic indeterminacy while maintaining mathematical coherence.

**Table 10:** Comparative Results of AES Models on Ambiguity- and Vagueness-Rich Benchmark Datasets.

Model	ASAP (2012)	ASAP-2.0	DREsS (EFL)	TOEFL11 Subset
Fuzzy Ontology Model	MAE = 0.68 *r* = 0.78	MAE = 0.72 *r* = 0.75	MAE = 0.80 *r* = 0.70	MAE = 0.77 *r* = 0.72
Type-1 Neutrosophic Ontology Model	MAE = 0.55 *r* = 0.85	MAE = 0.61 *r* = 0.82	MAE = 0.68 *r* = 0.76	MAE = 0.65 *r* = 0.78
Type-2 Neutrosophic Ontology (Proposed)	MAE = 0.43 *r* = 0.91	MAE = 0.52 *r* = 0.88	MAE = 0.55 *r* = 0.84	MAE = 0.58 *r* = 0.82

The observed performance gains indicate a stronger empirical alignment with human grading trends in linguistically ambiguous essays, rather than a definitive replication of human judgment or reasoning processes. In particular, the model does not possess the capacity for contextual interpretation, pedagogical intent recognition, or holistic reasoning that human raters employ when evaluating writing. Its improved alignment arises from enhanced statistical sensitivity to uncertainty and variability in surface-level linguistic and semantic features, not from an internalized understanding of meaning, intent, or evaluative rationale. Consequently, the model’s behavior reflects correlation with human scoring patterns under uncertainty, rather than equivalence to the cognitive or deliberative processes underlying human assessment.

To statistically confirm whether the proposed T2NS-based AES model significantly outperforms the baselines (Fuzzy and Type-1 Neutrosophic models), a one-way ANOVA was conducted for each benchmark dataset using MAE as the dependent variable and model type as the independent factor.

- Null Hypothesis ($\:H_{0}$): There is no significant difference in the mean MAE among the three AES models (Fuzzy, Type-1 Neutrosophic, and Type-2 Neutrosophic).
- Alternative Hypothesis ($\:H_{1}$): At least one model has a significantly different mean MAE compared to the others.

A significance level of α = 0.05 was used. Post-hoc pairwise comparisons (Tukey’s HSD) were applied when ANOVA results were significant.

**Table 11:** ANOVA statistics for model performance.

Dataset	Between-Group SS	Within-Group SS	F-value	*p*-value
ASAP (2012)	0.176	0.024	99.00	< 0.001
ASAP-2.0	0.141	0.028	67.96	< 0.001
DREsS (EFL)	0.158	0.035	60.86	< 0.001
TOEFL11 Subset	0.132	0.031	57.39	< 0.001

The ANOVA results in Table 11 clearly indicate that the differences in MAE across the three AES models—Fuzzy Ontology, Type-1 Neutrosophic Ontology, and 2NS—are statistically significant for all benchmark datasets. The F-values ranging from 57.39 to 99.00 demonstrate that the variation in performance is primarily driven by model type rather than random noise or dataset variability.

In particular, the largest F-value for the ASAP (2012) dataset (F = 99.00, *p* < 0.001) suggests that the effect of model design on predictive accuracy is especially pronounced in essays that contain nuanced linguistic ambiguity and mild figurative elements. This pattern holds across all datasets, confirming that the proposed T2NS framework consistently achieves lower MAE values, meaning it predicts human scores with higher precision under uncertain linguistic conditions.

From a linguistic and computational perspective, these ANOVA results demonstrate that the T2NS model’s hierarchical representation of uncertainty directly enhances interpretive robustness. While fuzzy logic captures partial truth values and Type-1 Neutrosophy introduces fixed degrees of indeterminacy, the Type-2 approach allows uncertainty intervals themselves to vary.

This flexibility enables the model to encode overlapping semantic meanings and linguistic vagueness with greater fidelity—aligning better with human evaluators who interpret essays through a similarly nuanced lens. The statistically significant reductions in MAE (*p* < 0.001) across all datasets thus confirm that the T2NS-enhanced AES pipeline not only produces quantitatively superior results but also achieves a more cognitively aligned representation of ambiguity and vagueness in natural language writing.

The Between-Group Sum of Squares (SS) values—ranging from 0.132 to 0.176. Higher Between-Group SS values, as seen in the ASAP (2012) and DREsS datasets, indicate that the choice of model architecture (Fuzzy, Type-1, or Type-2 Neutrosophic) accounts for a substantial proportion of the total variability in scoring performance.

This suggests that model design plays a decisive role in predictive accuracy. Conversely, the Within-Group SS values (0.024–0.035) are much smaller, implying that most of the observed variation in MAE cannot be attributed to random fluctuations within models. In other words, the results are stable across runs and dataset samples, reflecting strong computational consistency and reproducibility. The relatively small within-group variance confirms that the observed performance improvements stem from the models’ theoretical and structural differences rather than noise in the experimental setup.

The highest F-value observed for ASAP (2012) reflects this dataset’s sensitivity to ambiguity-handling capacity; here, the T2NS model demonstrates a dramatic reduction in MAE relative to other approaches. This suggests that T2NS particularly excels in cases where essays contain figurative expressions or semantic blending, both of which challenge conventional scoring logic. The p-values (< 0.001) across all datasets provide strong evidence against the null hypothesis (H₀), confirming that the differences among models are not due to random chance. These extremely small p-values reinforce the reliability of the T2NS model’s performance advantage.

**Table 12:** Tukey HSD Post-hoc Comparisons.

Dataset	Post-hoc (Tukey HSD)	Significant difference
ASAP (2012)	T2NS < Type-1 < Fuzzy	Yes
ASAP-2.0	T2NS < Type-1 < Fuzzy	Yes
DREsS (EFL)	T2NS < Type-1 < Fuzzy	Yes
TOEFL11 Subset	T2NS < Type-1 < Fuzzy	Yes

When examining the post-hoc Tukey HSD results, as shown in Table 12, the consistent ranking of T2NS < Type-1 < Fuzzy across all datasets reinforces the notion that introducing higher-order uncertainty modeling yields practical benefits. For example, in the DREsS (EFL) dataset—composed of essays by non-native speakers—the Type-2 system’s MAE (0.55) is significantly lower than that of the Fuzzy (0.80) and Type-1 (0.68) systems. This suggests that T2NS can better accommodate inconsistent grammar, ambiguous phrasing, and overlapping lexical cues that challenge conventional scoring algorithms.

Similarly, the TOEFL11 subset shows an improvement of nearly 25% in error reduction compared to the baseline Fuzzy model, further validating that multi-level uncertainty reasoning improves performance in linguistically diverse contexts. The Post-hoc (Tukey HSD) results further clarify the direction of improvement—consistently ranking T2NS < Type-1 < Fuzzy—showing that each layer of enhanced uncertainty representation contributes incremental but statistically meaningful performance gains. Finally, the “Significant Difference” column confirms that all datasets yield significance at α = 0.05, with consistent improvement patterns across heterogeneous linguistic contexts. This indicates that the T2NS model’s strength is not limited to a particular essay domain but generalizes well across diverse writing styles, including argumentative, EFL, and mixed-language compositions.

#### Experiment 3: Evaluation of fairness, transparency, and reliability

The objective of Experiment 3 is to examine whether the adaptive, context-aware scoring mechanism enabled by T2NS enhances fairness, transparency, and reliability when evaluating essays from diverse genres such as narrative, expository, and argumentative writing. The configuration involves scoring a mixed-genre dataset using two models: a static Type-1 ontology-based AES system and the proposed adaptive Type-2 T2NS model, which dynamically adjusts trait weightings and reasoning thresholds according to genre-specific linguistic and rhetorical patterns.

The evaluation compares both systems by analyzing scoring consistency and interpretability across genres. Three quantitative metrics are used: (1) Inter-Rater Reliability (IRR), computed using the QWK between automated predictions and multiple human raters to assess agreement and fairness; (2) Standard Deviation (SD) of automated scores across genres, where lower values indicate more stable and genre-consistent scoring behavior; and (3) a Transparency Index, providing a quantitative measure of interpretability.

In this experiment, fairness is interpreted operationally, as reflected by reduced score variance across genres, improved agreement metrics, and enhanced traceability of scoring factors, rather than as a universal or bias-free guarantee. Specifically, the proposed framework does not claim to eliminate all forms of demographic, cultural, linguistic, or prompt-specific bias, nor does it assert uniform fairness across all populations, writing styles, or assessment contexts. Instead, fairness is evaluated relative to measurable statistical indicators within the studied datasets, acknowledging that residual biases may persist and that broader normative, ethical, or societal notions of fairness fall outside the scope of this empirical analysis.

**Table 13:** Comparative Evaluation of Fairness, Transparency, and Reliability across Genre Types.

Model	Inter-Rater Reliability (QWK ↑)	Std. Dev. of Scores Across Genres (↓)	Transparency Index (%) ↑
Type-1 Ontology AES	0.82	0.093	68.5
Adaptive Type-2 T2NS AES	0.89	0.056	84.7

The results in Table 13 indicate that the proposed AES model achieves higher fairness and reliability in essay scoring compared to the traditional Type-1 ontology-based system. The Inter-Rater Reliability (QWK) between the automated scores and multiple human graders rises from 0.82 to 0.89, suggesting a significant improvement in alignment with human judgment.

This increase reflects the adaptive model’s capacity to contextualize linguistic and rhetorical variations across genres, ensuring that narrative creativity, argumentative logic, and expository clarity are each assessed with genre-appropriate criteria. The higher QWK values confirm that the adaptive scoring process reduces systemic bias toward particular writing styles—demonstrating fairer treatment of diverse authorial voices and more consistent alignment with expert human raters.

The Standard Deviation (SD) of scores across genres decreases notably from 0.093 to 0.056, providing strong evidence of enhanced genre consistency in the adaptive T2NS model. A lower SD implies that score variability between genres is substantially reduced, meaning that the system maintains stable scoring standards even when essay structures, topic forms, and rhetorical conventions differ.

This stability is largely due to the adaptive weighting mechanism in the Type-2 framework, which dynamically adjusts membership and non-membership intervals based on contextual linguistic cues. By accounting for genre-sensitive features such as narrative cohesion, argument density, or descriptive precision, the T2NS system avoids overfitting to one writing style and ensures a balanced evaluation standard—an essential quality for fairness and reliability in automated essay scoring.

The Transparency Index also improves markedly—from 68.5% in the Type-1 model to 84.7% in the adaptive T2NS model—highlighting a key strength of the proposed approach: interpretability. This metric reflects the proportion of scoring features whose contribution to the final score can be explicitly traced through ontology rules.

The substantial gain in transparency demonstrates that the T2NS model not only provides more accurate and consistent results but also enhances the explainability of the scoring process. Its multi-level uncertainty representation allows evaluators to identify why certain traits (e.g., argument strength or lexical richness) contributed to a specific score. Collectively, these findings show that the adaptive Type-2 framework achieves superior fairness, interpretability, and genre robustness, reinforcing its suitability for real-world educational assessment systems where transparency and equitable evaluation are essential.

#### Experiment 4: Comprehensive benchmark evaluation of accuracy, robustness, and interpretability

The goal of Experiment 4 is to rigorously validate the overall effectiveness and robustness of the proposed framework in comparison with traditional statistical and Type-1 ontology–based AES systems. This large-scale benchmark evaluation focuses on diverse datasets such as ASAP-AES and Kaggle AES, which encompass varied essay topics, genres, and linguistic complexities. The experiment configuration involves applying all models to the same benchmark datasets and comparing their predictive outputs in terms of accuracy, robustness, and interpretability.

The Statistical AES baseline serves as a conventional reference model that employs feature-engineering and regression-based prediction techniques. It uses handcrafted linguistic, syntactic, and lexical features—such as word count, average sentence length, part-of-speech ratios, and n-gram frequencies—to train a Multiple Linear Regression (MLR) model against human-assigned scores. While effective in structured or formulaic writing, this model treats essay components as independent features and lacks semantic reasoning or uncertainty modeling, making it less suited for ambiguous or context-dependent text.

To quantify comparative performance, MAE and Root Mean Squared Error (RMSE) are computed to measure deviation from human scores, while Pearson and Spearman correlations capture both linear and rank-order consistency with human ratings. In addition, robustness is analyzed by measuring model stability when scoring essays with figurative, uncertain, or multi-layered linguistic structures—highlighting sensitivity to semantic ambiguity.

Lastly, interpretability is quantified as the ratio of ontology-based feature contributions that can be explicitly traced to the final scoring decision, providing an empirical indicator of model transparency. Collectively, these measures provide a comprehensive assessment of how effectively the T2NS framework advances accuracy, fairness, and explainability in comparison to both data-driven statistical baselines and Type-1 ontology systems.

**Table 14:** Benchmark Comparison of AES Models on Full-Scale Datasets.

Model	MAE ↓	RMSE ↓	Pearson ↑	Spearman ↑	Robustness Score ↑	Interpretability (%) ↑
Statistical AES (Baseline)	0.74	0.89	0.78	0.75	0.68	42
Type-1 Ontology AES	0.61	0.76	0.85	0.83	0.78	64
**Type-2 Ontology AES** **(Proposed)**	**0.47**	**0.63**	**0.91**	**0.89**	**0.86**	**81**

The results in Table 14 reveal a consistent and substantial performance improvement achieved by the proposed model across all evaluation metrics. In terms of accuracy, the T2NS framework achieves the lowest MAE (0.47) and RMSE (0.63), outperforming both the statistical baseline and the Type-1 ontology model. This reduction in error indicates that the T2NS model more closely replicates human scoring patterns, particularly in essays containing figurative or ambiguous expressions. The improvement is attributable to its ability to handle second-order uncertainty, where not only the degree of truth but also the uncertainty in that truth value is explicitly modeled—leading to finer discrimination between subtle linguistic nuances.

From a correlation perspective, the Pearson (0.91) and Spearman (0.89) values demonstrate that T2NS achieves stronger linear and rank-order alignment with human judgments than the comparative models. The statistical AES system, though consistent in structured writing, lacks the semantic depth to account for contextual and rhetorical ambiguity, while the Type-1 ontology improves semantic grounding but remains constrained by single-layer uncertainty representation.

By contrast, the T2NS approach maintains consistent correlation across genres and linguistic variations, suggesting that it more effectively captures nonlinear relationships between discourse quality and scoring dimensions such as coherence, style, and argument strength. This stability in correlation underscores its ability to generalize across essay topics and writing proficiency levels.

Equally important, the robustness and interpretability metrics highlight the practical benefits of adopting T2NS in real-world AES systems. The robustness score of 0.86 indicates superior resilience when encountering essays with uncertain or blended meanings, showing that the model remains stable even under complex linguistic input. Moreover, the interpretability index of 81% surpasses both baselines, signifying that a larger proportion of scoring decisions are directly traceable to ontology-based reasoning rules.

This transparency facilitates human interpretability and pedagogical trust, providing clear explanations for score assignments. Overall, the collective rise across all metrics—particularly the dual gain in robustness and interpretability—validates that the T2NS framework achieves a balanced advancement in both performance and explainability, marking it as a significant step forward in the development of fair, transparent, and context-sensitive AES systems.

The consistent improvements over baseline models suggest robust performance within the evaluated datasets and prompts, while remaining bounded by the experimental scope and not asserting universal superiority across all AES scenarios. Specifically, the reported gains are confined to the datasets, prompt types, scoring rubrics, and linguistic conditions examined in this study, and may not directly generalize to unseen domains, alternative educational settings, low-resource languages, or substantially different essay genres. The proposed approach is therefore presented as an empirically validated enhancement under controlled conditions, rather than as a universally optimal solution for automated essay scoring across all tasks, populations, and deployment contexts.

#### Experiment 5: Sensitivity analysis of higher-order uncertainty

This experiment aims to evaluate how the inclusion of the FOU in the proposed model influences the stability and sensitivity of essay scoring under different uncertainty conditions. The configuration uses a curated subset of essays exhibiting diverse uncertainty levels—such as vague lexical choices, implicit reasoning, or mixed sentiment—to assess how model predictions respond to varying degrees of indeterminacy. The experimental procedure involves scoring these essays using two system configurations: one employing the full FOU-based Type-2 neutrosophic reasoning and another simplified version where second-order uncertainty propagation is disabled.

The simplified version, where second-order uncertainty propagation FOU is disabled, is implemented by collapsing each Type-2 neutrosophic interval into single scalar values—typically by taking their midpoints—thereby converting the model into a Type-1 neutrosophic framework that performs all reasoning, aggregation, and defuzzification using deterministic scalar operations instead of interval-based computations, thus eliminating uncertainty spread while preserving the same ontology structure and rule base for direct sensitivity comparison. The analysis then focuses on quantifying how score outputs change as the FOU parameters (i.e., the upper and lower bounds of membership and indeterminacy intervals) are systematically varied.

In our case, the curated subset was obtained through a controlled filtering strategy aimed at maximizing the presence of uncertainty phenomena. Essays were included if they satisfied at least one of the following quantitative indicators: (i) high semantic dispersion among sentence embeddings (upper quartile of intra-essay cosine variance), signaling argument fragmentation or topical blending; (ii) elevated lexical ambiguity estimated through WordNet synset multiplicity or disagreement among contextual embeddings; (iii) presence of hedging or stance-modulating expressions detected using a predefined list of linguistic markers; and (iv) in EFL datasets, atypical grammar-to-content error ratios suggesting semantic opacity rather than purely mechanical mistakes.

Thresholds were computed independently for each prompt to prevent topic bias and to respect differences in writing style, length, and rubric expectations. Concretely, for a given prompt p, distributions of each ambiguity indicator were first calculated over the full training portion of that prompt. Selection cutoffs were then derived using percentile statistics.

For example, in ASAP Prompt 3, the intra-essay cosine variance ranged from 0.08 to 0.41 with a 75th-percentile value of 0.29; essays with variance ≥ 0.29 were therefore marked as semantically fragmented. Similarly, if the median number of hedging expressions for that prompt was 4 per essay, submissions exceeding this value were flagged as containing elevated stance uncertainty. In TOEFL11, grammar-to-content ratios above the prompt-specific 70th percentile (e.g., > 1.6) were treated as indicators of semantic opacity.

By calibrating thresholds locally rather than globally, the procedure avoids unfairly labeling naturally complex prompts as ambiguous while still isolating essays where uncertainty modeling is most critical. The resulting subset therefore concentrates challenging uncertainty cases while maintaining score distributions comparable to the original corpora.

Two main metrics are applied: (1) score deviation relative to human scores, calculated as the mean absolute change between automated and reference ratings before and after FOU adjustments, and (2) a sensitivity index, defined as the percentage variation in predicted scores for incremental shifts in FOU width (e.g., ± 5–20%). Lower sensitivity index values indicate greater scoring stability and robustness, while controlled deviations highlight the FOU’s role in modeling uncertainty more realistically without amplifying noise. Together, these analyses reveal the balance between interpretability and numerical stability in higher-order neutrosophic representations, demonstrating how second-order uncertainty modeling refines the precision and adaptability of automated essay scoring.

**Table 15:** Sensitivity Analysis of Higher-Order Uncertainty.

Model Configuration	Mean Score Deviation (Δ from Human)	Sensitivity Index (%)	Stability Indicator
Type-1 Neutrosophic (FOU Disabled)	0.64	12.8	Moderate
Type-2 Neutrosophic (FOU Enabled, ± 5%)	0.48	7.5	High
Type-2 Neutrosophic (FOU Enabled, ± 10%)	0.50	8.1	High
Type-2 Neutrosophic (FOU Enabled, ± 20%)	0.54	9.4	High

The results in Table 15 clearly demonstrate that incorporating the FOU into the Type-2 Neutrosophic Ontology significantly enhances the stability and precision of essay scoring outcomes. The mean score deviation (MAE-like measure) between automated and human ratings drops from 0.64 in the Type-1 configuration to 0.48–0.54 in the Type-2 configurations, indicating that the Type-2 model produces scores that align more closely with human judgments even when dealing with essays that exhibit vague or implicit linguistic structures. This improvement underscores how second-order uncertainty representation allows the model to flexibly capture semantic nuances that Type-1 reasoning oversimplifies, reducing systematic bias caused by ambiguous or context-dependent features.

When examining the sensitivity index, the Type-2 FOU-enabled system exhibits substantially lower sensitivity (7.5–9.4%) compared to the Type-1 baseline (12.8%), meaning its predictions remain relatively stable even as FOU parameters are perturbed by ± 5–20%. This lower sensitivity reflects the model’s robustness to small fluctuations in uncertainty bounds, a desirable property in automated assessment since minor linguistic variations or grader subjectivity should not produce disproportionately large shifts in the final score. The slight increase in sensitivity at higher FOU variations (e.g., ± 20%) demonstrates controlled adaptability rather than instability—showing that the model remains flexible enough to respond to genuine uncertainty without overreacting to noise.

The sensitivity analysis shows that modeling higher-order uncertainty through FOU tends to reduce score volatility under controlled conditions, indicating improved robustness rather than absolute stability. In this context, absolute stability would imply invariant scoring behavior across all levels of linguistic noise, prompt variation, and feature perturbation—an assumption that is neither realistic nor claimed in this work. Instead, the observed robustness reflects a relative dampening of score fluctuations in response to uncertainty, acknowledging that performance may still vary under extreme ambiguities, distributional shifts, or conditions beyond those explicitly modeled in the experimental setup.

Overall, these findings validate the superiority of the Type-2 FOU-enabled T2NS model in balancing interpretability, adaptability, and reliability under uncertain linguistic conditions. The consistent reduction in deviation and sensitivity highlights how higher-order uncertainty propagation refines the model’s capacity to reason under vagueness while maintaining coherent scoring behavior. In practical terms, this means the T2NS system can fairly and consistently assess essays with figurative, imprecise, or context-rich language—an area where traditional and Type-1 AES systems often fail. This robustness and stability establish the Type-2 neutrosophic ontology as a powerful framework for next-generation AES systems that must handle the inherent ambiguity of natural language in educational contexts.

#### Experiment 6: Benchmark comparison with transformer-based AES models

The objective of this experiment is to benchmark the proposed framework against recent transformer-based neural AES systems in order to evaluate its competitive performance relative to current state-of-the-art approaches. Specifically, the experiment examines whether the proposed uncertainty-aware ontology model can achieve comparable predictive accuracy while providing advantages in robustness and interpretability.

To this end, four representative transformer-based AES methods from recent literature are used as comparative baselines: the Hierarchical BERT-based AES model^49^, which uses hierarchical sentence- and document-level contextual representations; the Transformer-based AES framework^50^, which applies a standard transformer encoder with a regression scoring layer; the Transformer–LSTM hybrid model^51^, which combines transformer embeddings with sequential LSTM modeling; and the ET-GNN model^52^, which integrates transformer semantic encoding with graph neural network structures to capture essay feature relationships.

Each comparative model was implemented according to the configuration described in the corresponding studies and fine-tuned on the same TOEFL11 dataset, ensuring consistent training–validation–testing splits across all approaches. Transformer models were fine-tuned using a regression-based scoring setup, while the proposed T2NS framework applied ontology reasoning combined with interval-valued neutrosophic uncertainty modeling for trait-level aggregation. The evaluation employed widely accepted AES metrics, including QWK, MAE, RMSE, and Pearson correlation. In addition, the experiment reports Robustness Score and Transparency/Interpretability indices to highlight differences between neural black-box models and the proposed interpretable uncertainty-aware framework.

The results in Table 16 indicate that the proposed framework consistently outperforms the transformer-based models across multiple key metrics. In terms of predictive agreement with human raters, measured by QWK, T2NS achieves a score of 0.87, surpassing the ET-GNN model (0.84) and other transformer baselines (0.80–0.83). This higher QWK demonstrates that the proposed framework aligns more closely with human judgment, particularly in essays exhibiting linguistic ambiguity or figurative language, where conventional transformer models may fail to capture subtle semantic details.

**Table 16:** Comparative Performance of T2NS AES vs. Transformer-Based AES Models on TOEFL11 dataset.

Model	QWK ↑	MAE ↓	RMSE ↓	Pearson ↑	Robustness Score ↑	Interpretability (%) ↑
Hierarchical BERT AES^49^	0.82	0.57	0.73	0.86	0.74	45
Transformer-based AES^50^	0.80	0.59	0.75	0.84	0.72	43
Transformer–LSTM Hybrid AES^51^	0.83	0.56	0.72	0.87	0.76	44
ET-GNN AES^25^	0.84	0.54	0.70	0.88	0.77	46
**Proposed Type-2 Neutrosophic AES (T2NS)**	**0.87**	**0.47**	**0.63**	**0.91**	**0.86**	**81**

Regarding error measures, T2NS also shows clear advantages. The MAE and RMSE for T2NS are 0.47 and 0.63, respectively, which are substantially lower than the transformer-based models (MAE = 0.54–0.57, RMSE = 0.70–0.75). These reductions indicate that the proposed framework predicts essay scores more accurately, reflecting its ability to handle uncertainty, indeterminacy, and overlapping semantic cues through interval-valued neutrosophic reasoning. Similarly, the Pearson correlation of 0.91 demonstrates superior linear alignment with human scores, exceeding the best-performing transformer model (ET-GNN, 0.88), which suggests improved consistency in capturing the relationships between essay features and overall score.

Beyond accuracy, T2NS exhibits notable advantages in robustness and interpretability, two areas where black-box transformer models are limited. The Robustness Score of 0.86 indicates that T2NS predictions remain stable under variations in essay content, linguistic ambiguity, and mixed-language elements, outperforming transformer models (0.72–0.77). This reflects the framework’s capacity to explicitly represent uncertainty at the trait level, mitigating volatility caused by ambiguous or complex inputs.

Moreover, T2NS achieves an Interpretability index of 81%, nearly double that of transformer models (43–46%), demonstrating that a large proportion of the scoring decisions can be traced to ontology rules and trait-level uncertainty intervals. This transparency allows educators and evaluators to inspect the contribution of individual essay traits to the final score, a feature that transformer-based models cannot provide.

Collectively, these results indicate that the proposed T2NS AES framework delivers balanced improvements across predictive accuracy, robustness, and interpretability, addressing both quantitative and qualitative aspects of automated essay scoring. While transformer models are effective in capturing semantic embeddings and document-level context, they remain opaque and less robust under uncertainty^53,54^. In contrast, the proposed uncertainty-aware ontology approach leverages higher-order neutrosophic modeling to provide a scoring system that is not only accurate but also reliable, explainable, and adaptable to diverse essay types and linguistic complexities, highlighting its practical advantages for real-world educational assessment.

#### Experiment 7: Ablation analysis of the type-2 neutrosophic ontology framework

The objective of this experiment is to analyze the individual contribution of the main components of the proposed framework and to determine how each module influences the overall scoring performance. In particular, the study investigates the effect of Type-2 neutrosophic uncertainty modeling, the performance difference between Type-1 and Type-2 neutrosophic representations, and the role of spatial autocorrelation features in capturing dependencies among essay traits. By progressively removing or replacing specific components, the experiment aims to quantify the added value of each element and verify whether the integration of these modules leads to measurable improvements in scoring accuracy, robustness, and interpretability.

To achieve this, several ablation variants of the framework were implemented. These include: (1) an ontology-based baseline model that performs concept extraction and rule-based scoring without neutrosophic uncertainty modeling, (2) a Type-1 neutrosophic variant (Ontology + T1NS) that introduces basic neutrosophic membership functions, (3) a spatially enhanced ontology model (Ontology + Spatial Autocorrelation) that incorporates trait dependency analysis, (4) a Type-2 neutrosophic model without spatial features (Ontology + T2NS), and (5) the full proposed framework (Ontology + T2NS + Spatial Autocorrelation).

All variants were evaluated on the TOEFL11 dataset using the same preprocessing pipeline, ontology structure, and experimental settings to ensure a controlled comparison. Performance was measured using widely adopted AES evaluation metrics, including QWK to assess agreement, MAE and RMSE, Pearson correlation, and a robustness indicator to assess model stability under linguistic variability. These metrics collectively provide a comprehensive evaluation of the contribution of each component to the effectiveness of the proposed AES framework.

**Table 17:** Ablation Study of the Proposed T2NS AES Framework (TOEFL11).

Model Variant	QWK ↑	MAE ↓	RMSE ↓	Pearson ↑	Robustness ↑
Ontology Baseline (Concept + Rules)	0.79	0.60	0.76	0.84	0.71
Ontology + Type-1 Neutrosophic (T1NS)	0.82	0.56	0.72	0.87	0.76
Ontology + Spatial Autocorrelation	0.81	0.58	0.74	0.86	0.78
Ontology + Type-2 Neutrosophic (T2NS)	0.85	0.50	0.67	0.89	0.83
**Full Model (Ontology + T2NS** **+ Spatial Autocorrelation)**	**0.87**	**0.47**	**0.63**	**0.91**	**0.86**

The results in Table 17 demonstrate the impact of each component of the proposed framework on automated essay scoring performance. The ontology baseline model, which relies solely on concept extraction and rule-based reasoning, achieves a QWK of 0.79, MAE of 0.60, and RMSE of 0.76. While this configuration already provides interpretable scoring decisions through semantic rules, its performance is relatively limited because it lacks mechanisms to explicitly represent uncertainty and trait interactions in essay content. Consequently, the robustness score remains relatively low (0.71), indicating sensitivity to linguistic ambiguity and variations in writing style.

Introducing Type-1 neutrosophic modeling (Ontology + T1NS) improves performance across all metrics. The QWK increases to 0.82, while MAE and RMSE decrease to 0.56 and 0.72, respectively. This improvement occurs because Type-1 neutrosophic sets allow the model to represent degrees of truth, falsity, and indeterminacy when evaluating essay traits. As a result, the model can better handle vague or partially relevant concepts in student essays. However, since Type-1 neutrosophic representations use fixed membership values, their ability to capture higher-order uncertainty remains limited compared with Type-2 modeling.

The variant incorporating spatial autocorrelation features (Ontology + Spatial) achieves a QWK of 0.81 and improves the robustness score to 0.78. This component captures dependencies among essay traits, such as the relationships between topic relevance, coherence, and argument development. By modeling these dependencies, the system becomes more stable when evaluating essays where multiple traits interact. However, because this variant still lacks advanced uncertainty modeling, its gains in predictive accuracy (MAE and RMSE) remain moderate.

A more substantial improvement is observed when Type-2 neutrosophic modeling (Ontology + T2NS) is introduced. This configuration achieves QWK = 0.85, MAE = 0.50, and RMSE = 0.67, significantly outperforming both the baseline and Type-1 variants. Type-2 neutrosophic sets represent uncertainty using interval-valued membership functions, allowing the model to capture the variability and ambiguity inherent in essay language. This richer uncertainty representation leads to more reliable scoring decisions and a higher robustness score (0.83), indicating improved stability across essays with diverse linguistic characteristics.

The full proposed framework (Ontology + T2NS + Spatial Autocorrelation) achieves the best performance across all evaluation metrics. The model reaches QWK = 0.87, MAE = 0.47, RMSE = 0.63, and Pearson correlation = 0.91, while the robustness score increases to 0.86. These results demonstrate that combining Type-2 neutrosophic uncertainty modeling with spatial trait relationships produces a synergistic effect. The neutrosophic component effectively captures linguistic uncertainty, while the spatial autocorrelation mechanism models interactions among essay traits. Together with ontology-based semantic reasoning, these components enable the system to produce more accurate, robust, and interpretable scoring outcomes.

Overall, the ablation study confirms that each component contributes positively to the framework’s performance, with Type-2 neutrosophic modeling providing the largest gain in accuracy, and spatial autocorrelation improving robustness and stability. The integration of these elements in the final architecture explains the superior performance of the proposed model compared with simpler configurations.

### Experimental comparability and interpretability–performance trade-off

To ensure the validity and fairness of the comparative analysis, all baseline models—including the statistical AES model, the Type-1 ontology-based system, and the transformer-based neural models—were evaluated under strictly controlled and identical experimental conditions. Specifically, all approaches were trained and tested using the same datasets and standardized training/validation/testing splits, while identical preprocessing pipelines were applied across models to eliminate data-related biases. Furthermore, a unified set of evaluation metrics, including QWK, MAE, RMSE, and Pearson correlation, was consistently employed to ensure comparability of results.

For neural baselines, transformer-based models were re-implemented and fine-tuned using the same dataset partitions (e.g., TOEFL11) and regression-based scoring configurations, thereby ensuring that performance differences are attributable to modeling strategies rather than discrepancies in experimental setup. Similarly, ontology-based variants share an identical knowledge base, and feature extraction process, differing only in their treatment of uncertainty (Type-1 versus Type-2 neutrosophic representations). This controlled design guarantees that the reported improvements stem directly from the proposed uncertainty modeling framework.

The experimental results also highlight a fundamental trade-off between predictive performance and interpretability in AESs. Transformer-based neural models demonstrate strong representation learning capabilities and achieve competitive accuracy; however, they operate as black-box systems, providing limited insight into how specific linguistic or semantic features influence the final score. In contrast, ontology-based approaches offer inherently transparent and explainable reasoning through explicit rule-based structures, yet they have traditionally been constrained in handling complex linguistic uncertainty.

The proposed Type-2 Neutrosophic framework effectively bridges this gap by achieving a balanced trade-off between these two dimensions. While maintaining competitive predictive performance relative to transformer-based models, the framework significantly enhances interpretability, achieving transparency levels of approximately 81%. This improvement is primarily attributed to the explicit modeling of higher-order uncertainty through interval-valued neutrosophic representations, rather than an increase in model complexity alone. As a result, the system not only produces accurate predictions but also provides traceable and interpretable justifications for scoring decisions.

From a broader perspective, the proposed T2NS-based AES framework can be viewed as a hybrid paradigm that integrates the strengths of symbolic and data-driven approaches. Unlike purely neural models, which prioritize predictive power at the expense of explainability, and traditional ontology-based systems, which emphasize interpretability but lack flexibility, the proposed approach combines structured semantic reasoning with advanced uncertainty modeling.

It is important to note that the framework is not intended to universally replace neural AES models. Instead, it is positioned as a complementary and practically advantageous solution for scenarios where transparency, fairness, and robustness are critical requirements—particularly in educational contexts. By enabling explicit reasoning over uncertain linguistic features and providing interpretable scoring pathways, the proposed model supports educationally meaningful assessment, aligning with the increasing demand for explainable artificial intelligence in e-learning systems.

### Implications of Type-2 Neutrosophic Ontology–Based AES for E-Education and Intelligent Learning Systems

The integration of the proposed model into e-education and e-learning systems carries significant implications for intelligent assessment, adaptive feedback, and data-driven decision support. By modeling higher-order uncertainty, the T2NS framework enables more equitable and context-aware essay evaluation, ensuring that students are graded fairly regardless of linguistic style, cultural background, or writing genre. Its interpretability allows educators and learning analytics systems to trace scoring rationales, transforming the AES from a black-box grader into a transparent pedagogical assistant.

In e-learning environments, this capability supports personalized feedback loops, where students receive constructive insights about their strengths and areas of ambiguity rather than mere numeric scores. Furthermore, the integration of T2NS reasoning into institutional decision support systems can enhance curriculum planning, formative assessment, and learner modeling by providing reliable, uncertainty-aware evaluations at scale.

### Feasibility analysis

The feasibility study of the proposed framework demonstrates that its integration into real-world educational systems is both technically and operationally viable. From a computational perspective, the ontology-driven structure ensures modular scalability, allowing seamless incorporation into existing Learning Management Systems (LMS) and e-assessment platforms without major architectural overhauls. The use of Type-2 neutrosophic reasoning, while more computationally intensive than Type-1 systems, remains practical with modern processing capabilities and can be optimized through parallel inference and fuzzy interval approximation techniques.

Empirical evaluations on benchmark datasets confirm the model’s robustness, reliability, and interpretability, supporting its adaptability across diverse essay topics and writing genres. Moreover, its transparent rule-based structure aligns well with educational policy standards for fairness and explainability, ensuring institutional acceptance. Overall, the feasibility study underscores that deploying the T2NS-based AES framework in e-learning environments is both realistic and sustainable, offering measurable improvements in assessment quality, transparency, and pedagogical value.

### Limitations

Despite its demonstrated advantages, the proposed model faces several limitations that warrant further investigation. First, the computational complexity introduced by the higher-order uncertainty representation and the FOU expansion increases processing time, which may hinder real-time deployment in large-scale educational systems without optimization. Second, while the ontology-driven structure enhances interpretability, its initial construction and continuous maintenance demand expert knowledge in both linguistics and ontology engineering, making scalability and domain adaptation resource-intensive.

Additionally, the model’s performance still partially depends on the quality and diversity of training data—limited exposure to unconventional writing styles, creative narratives, or non-native linguistic patterns may reduce generalization. Another challenge lies in balancing sensitivity and stability; overly wide FOU intervals can lead to interpretive ambiguity, while narrow intervals may suppress subtle linguistic nuances. Finally, although the framework improves transparency, translating its neutrosophic reasoning outcomes into easily understandable feedback for students and instructors remains a complex task that requires user-centered interface design and educational validation.

## Conclusions

This study introduced a Type-2 Neutrosophic Ontology–based AES framework, a novel approach designed to address the persistent challenge of uncertainty, vagueness, and ambiguity in linguistic evaluation. The proposed methodology extends traditional ontology-based AES by embedding a higher-order uncertainty representation through the FOU, enabling the model to reason about both uncertainty and the uncertainty of uncertainty itself.

This dual-layer reasoning allows for nuanced handling of figurative language, implicit argumentation, and overlapping semantic constructs that conventional Type-1 and statistical AES models struggle to interpret. Empirical evaluations conducted across multiple benchmark datasets—including ASAP, DREsS (EFL), and TOEFL11—demonstrated the superiority of the T2NS framework, achieving the lowest MAE (≈ 0.43–0.55), highest Pearson correlation (≈ 0.88–0.91), and significant improvements in ANOVA performance (*p* < 0.001).

Furthermore, the adaptive scoring mechanism enhanced fairness and transparency, yielding higher inter-rater reliability and consistent performance across diverse genres. Collectively, these results affirm that the integration of Type-2 neutrosophic reasoning marks a substantial methodological advancement in intelligent essay evaluation, bridging the gap between symbolic interpretability and data-driven precision.

Looking ahead, future work will focus on enhancing the scalability and adaptability of the proposed model within large-scale e-learning ecosystems. Optimization of computational efficiency through hybrid fuzzy–neutrosophic approximation and parallelized inference will enable real-time deployment in cloud-based learning platforms.

Additionally, future studies will explore cross-linguistic generalization, applying the framework to multilingual essay datasets and discipline-specific writing tasks to assess its robustness in varied educational contexts. Another promising direction involves the integration of explainable AI (XAI) visualization modules to transform neutrosophic reasoning outputs into interpretable feedback for students and instructors, thereby deepening pedagogical value. By continuing to refine uncertainty modeling and adaptive reasoning, the T2NS-based AES framework stands poised to revolutionize automated assessment—making it more human-like, equitable, and trustworthy within modern digital education systems.

## Electronic Supplementary Material

Below is the link to the electronic supplementary material.

Supplementary Material 1

## Data Availability

The datasets generated and/or analyzed during the current study are available in the Kaggle repository, https://www.kaggle.com/competitions/learning-agency-lab-automated-essay-scoring-2.

## Code availability

**Statement**: The Python implementation of the proposed Type-2 Neutrosophic Ontology-based Automated Essay Scoring framework is provided as supplementary material accompanying this manuscript. The code package includes a detailed README file with step-by-step instructions for environment setup, data preprocessing, model training, and evaluation to ensure full reproducibility of the reported results.

## Declarations

### Supplementary Information

The Python implementation of the proposed Type-2 Neutrosophic Ontology-based Automated Essay Scoring framework is provided as supplementary material accompanying this manuscript.

## References

[1] Link, S. & Koltovskaia, S. Automated Scoring of Writing. In *Digital Writing Technologies in Higher Education: Theory, Research, and Practice* 333–345 (Springer International Publishing, 2023).

[2] Ramesh, D. & Sanampudi, S. An automated essay scoring systems: a systematic literature review. *Artif. Intell. Rev.* **55** (3), 2495–2527 (2022). 34584325 10.1007/s10462-021-10068-2 PMC8460059

[3] Honko, M., Neittaanmäki, R., Jarvis, S. & Huhta, A. Beyond literacy and competency–The effects of raters’ perceived uncertainty on assessment of writing. *Assess. Writ.* **57** (6), 1–14. 100768 (2023).

[4] Huawei, S. & Aryadoust, V. A systematic review of automated writing evaluation systems. *Educ. Inform. Technol.* **28** (1), 771–795 (2023).

[5] Susanti, M., Ramadhan, A. & Warnarsa, H. Automatic essay exam scoring system: a systematic literature. *Procedia Comput. Sci.* **216** (1), 531–538 (2023). 36643175 10.1016/j.procs.2022.12.166 PMC9829427

[6] Shang, W., Men, H., Du, X. & Machine Learning Models A Study of English Essay Text Content Feature Extraction and Automatic Scoring. *J. ICT Stand.* **11** (4), 379–390 (2023).

[7] Ajetunmobi, S. & Daramola, O. Ontology-based information extraction for subject-focused automatic essay evaluation. In *Proceedings of the International Conference on Computing Networking and Informatics*, pp. 1–6, IEEE, (2017).

[8] Darwish, S. & Mohamed, S. Automated essay evaluation based on fusion of fuzzy ontology and latent semantic analysis. In *Proceedings of the International Conference on Advanced Machine Learning Technologies and Applications*, pp. 566–575, Springer International Publishing, (2020).

[9] Contreras, J., Hilles, S. & Abubakar, Z. Automated essay scoring using ontology generator and natural language processing with question generator based on blooms taxonomy’s cognitive level. *Int. J. Eng. Adv. Technol.* **9** (1), 2448–2457 (2019).

[10] Almuayqil, S., El-Ghany, A. & Shehab, S. Towards an Ontology-Based Fully Integrated System for Student E-Assessment. *J. Theoretical Appl. Inform. Technol.* **98** (21), 3514–3527 (2020).

[11] Hernández, N. et al. Model based on neutrosophic ontologies for the study of entrepreneurship competence. *Neutrosophic Sets Syst.* **51** (1), 924–928 (2022).

[12] Bhutani, K. & Aggarwal, S. Experimenting with neutrosophic ontologies for medical data classification. In *Proceedings of the IEEE Workshop on Computational Intelligence: Theories, Applications and Future Directions*, pp. 1–6, (2015).

[13] Darwish, S., Ali, R. & Elzoghabi, A. An Automated English Essay Scoring Engine Based on Neutrosophic Ontology for Electronic Education Systems. *Applied Sciences*, **13**(6), 1–26, 8601, 2023.

[14] Zolfani, S., Görçün, Ö., Çanakçıoğlu, M. & Tirkolaee, E. Efficiency analysis technique with input and output satisficing approach based on Type-2 Neutrosophic Fuzzy Sets: A case study of container shipping companies. *Expert Syst. Applications* **218**(1), 1–21, 119596, 2023.

[15] Hassan, M., Darwish, S. & Elkaffas, S. Type-2 Neutrosophic Set and Their Applications in Medical Databases Deadlock Resolution. *Computers Mater. Continua*. **74** (2), 4417–4434 (2023).

[16] Uto, M. A review of deep-neural automated essay scoring models. *Behaviormetrika* **48** (2), 459–484 (2021).

[17] Lim, C., Bong, C., Wong, W. & Lee, N. A comprehensive review of automated essay scoring (AES) research and development. *Pertanika J. Sci. Technol.* **29** (3), 1875–1899 (2021).

[18] Arbaaeen, A. & Shah, A. Ontology-based approach to semantically enhanced question answering for closed domain: A review. *Information*, **12**(5), 1 –25, 200, 2021.

[19] Srivastava, K., Dhanda, N. & Shrivastava, A. An analysis of automated essay grading systems. *Int. J. Recent. Technol. Eng.* **8** (6), 5438–5441 (2020).

[20] Ramnarain-Seetohul, V., Bassoo, V. & Rosunally, Y. Similarity measures in automated essay scoring systems: A ten-year review. *Educ. Inform. Technol.* **7** (4), 5573–5604 (2022).

[21] Goura, V., Moulesh, M., Madhusudanarao, N. & Gao, X. An efficient and enhancement of recent approaches to build an automated essay scoring system. *Procedia Comput. Sci.* **215** (2), 442–451 (2022).

[22] Hadyaoui, A. & Cheniti-Belcadhi, L. Towards an Ontology-based Recommender System for Assessment in a Collaborative ELearning Environment. In *Proceeding of the 18th International Conference on Web Information Systems and Technologies*, pp. 294–301, (2022).

[23] Contreras, J., Hilles, S. & Abubakar, Z. Automated essay scoring with ontology based on text mining and NLTK tools. In Proceedings of the International Conference on Smart Computing and Electronic Enterprise, pp. 1–6, IEEE, (2018).

[24] Leo, J. et al. Ontology-based generation of medical, multi-term MCQs. *Int. J. Artif. Intell. Educ.* **29** (4), 145–188 (2019).

[25] Radovic, M., Petrovic, N. & Tosic, M. An ontology-driven learning assessment using the script concordance test. *Applied Sciences* **12**(3), 1–24, 1472, 2022.

[26] Ramnarain-Seetohul, V., Bassoo, V. & Rosunally, Y. O. B. M. A. A. Q. Ontology-Based Model for Automated Assessment of Short-Answer Questions. In Proceedings of the First International Conference on Advances in Electrical, Electronics and Computational Intelligence, pp. 1–8, (2023).

[27] Beck, M., Rizvi, S., Dengel, A. & Ahmed, S. From automatic keyword detection to ontology-based topic modeling. In *Proceedings of the International workshop on document analysis systems*, pp. 451–465, Cham: Springer International Publishing, (2020).

[28] Premathilaka, Y., Banujan, K. & Kumara, B. Ontology-Based Approach to Determine the Coverage of Examination Papers. In Proceedings of the International Conference on Decision Aid Sciences and Application, pp. 613–617, IEEE, (2020).

[29] Hadyaoui, A. & Cheniti-Belcadhi, L. Ontology-based group assessment analytics framework for performances prediction in project-based collaborative learning. *Smart Learn. Environ.* **10** (1), 1–27 (2023). 43.

[30] Bakali, A. et al. Graphical representation of type-2 neutrosophic sets. *Neutrosophic Sets Syst.* **42** (3), 28–38 (2021).

[31] Singh, P. A type-2 neutrosophic-entropy-fusion based multiple thresholding method for the brain tumor tissue structures segmentation. *Appl. Soft Comput.* **103** (1), 1–23 (2021). 107119.

[32] Nandini, V. & Maheswari, P. U. Automatic assessment of descriptive answers in online examination system using semantic relational features. *J. Supercomputing*. **76** (3), 4430–4448 (2020).

[33] Hoblos, J. Experimenting with latent semantic analysis and latent Dirichlet allocation on automated essay grading. In *Proceedings of the Seventh International Conference on Social Networks Analysis, Management and Security*, Paris, France, 14–16 December, pp. 1–7, (2020).

[34] Chan, K., Bond, T. & Yan, Z. Application of an automated essay scoring engine to English writing assessment using many-facet rasch measurement. *Lang. Test.* **40** (1), 61–85 (2023).

[35] Verdikha, N., Thamrin, H., Triyono, A., Abdillah, M. & Suryawan, S. Regression and oversampling method for Indonesian language automated essay scoring. In *Proceedings of the AIP Conference*, Vol. 2727, No. 1, AIP Publishing, (2023).

[36] Polak, J. & Cook, D. A study on student performance, engagement, and experience with Kaggle in class data challenges. *J. Stat. Data Sci. Educ.* **29** (2), 63–70 (2021).

[37] Hannah, L., Jang, E., Shah, M. & Gupta, V. Validity Arguments for Automated essay scoring of young students’ writing traits. *Lang. Assess. Q.* **20** (4–5), 399–420 (2023).

[38] Wilson, J. & Roscoe, R. D. Automated writing evaluation and feedback: Multiple metrics of efficacy. *J. Educational Comput. Res.* **58** (1), 87–125 (2020).

[39] Sireesha, V., Hegde, N., Kumar, S., Naravajhula, A. & Haritha, D. Automatic Essay Grading System Using Deep Neural Network. In *Proceedings of the International Conference on Information and Management Engineering*, pp. 537–544, Singapore: Springer Nature Singapore, (2022).

[40] Ramnarain-Seetohul, V., Bassoo, V. & Rosunally, Y. Similarity measures in automated essay scoring systems: A ten-year review. *Educ. Inform. Technol.* **27** (4), 5573–5604 (2022).

[41] Jiang, Z. et al. Learning from graph propagation via ordinal distillation for one-shot automated essay scoring. In Proceedings of the Web Conference, pp. 2347–2356, (2021).

[42] Sarwar, S., Qayyum, Z., García-Castro, R., Safyan, M. & Munir, R. Ontology based E-learning framework: A personalized, adaptive and context aware model. *Multimedia Tools Appl.* **78** (24), 34745–34771 (2019).

[43] Shah, N. & Pareek, J. Automatic evaluation of free text answers: A review. In *Proceedings of the International Conference on Advancements in Smart Computing and Information Security*, pp. 232–249, Cham: Springer Nature Switzerland, (2022).

[44] Mizumoto, A. & Eguchi, M. Exploring the potential of using an AI language model for automated essay scoring. *Research Methods Appl. Linguistics*, **2**(2), 1–13, 100050, 2023.

[45] Bai, X. & Stede, M. A survey of current machine learning approaches to student free-text evaluation for intelligent tutoring. *Int. J. Artif. Intell. Educ.* **33** (4), 992–1030 (2023). 10.1007/s40593-022-00323-0 PMC9707071 36467629

[46] Li, J. & Wu, J. Automated essay scoring incorporating multi-level semantic features. In *Proceedings of the International Conference on Artificial Intelligence in Education*, pp. 206–211, Cham: Springer Nature Switzerland, (2023).

[47] Vijaya, S., Guruvyas, K., Patil, P. & Acharya, J. Essay scoring systems using AI and feature extraction: A review. *In Proceedings of third international conference on communication, computing and electronics systems*, pp. 45–57, Singapore: Springer Singapore, (2022).

[48] Gandon, F. Q2Forge: Minting Competency Questions and SPARQL Queries for Question-Answering Over Knowledge Graphs. arXiv preprint arXiv:2505, 13572, 74-81 (2025).

[49] Xue, J., Tang, X. & Zheng, L. A hierarchical BERT-based transfer learning approach for multi-dimensional essay scoring. *Ieee Access.* **9**, 125403–125415 (2021).

[50] Chavva, R. K. R., Muthyam, S. R., Seelam, M. S. & Nalliboina, N. A Transformer-Based Approach for Enhancing Automated Essay Scoring. In 2024 1st International Conference on Advanced Computing and Emerging Technologies (ACET) (1–6). IEEE. (2024).

[51] Xuan, Y. *Transformer-LSTM Models for Automatic Scoring and Feedback in English Writing Assessment* (IEEE Access, 2025).

[52] Aljuaid, H., Alhothali, A., Alzamzami, O., Assalahi, H. & Aldosemani, T. *ET-GNN: ensemble transformer-based graph neural networks for holistic automated essay scoring* (IEEE Access, 2025).

[53] Elmassry, A. M., Zaki, N., Alsheikh, N. & Mediani, M. *A systematic review of pretrained models in automated essay scoring* (IEEE Access, 2025).

[54] Kadimova, D., Azizova, B., Mengliyev, I. & Mamatov, J. Analysis of deep learning models for automated essay scoring. In AIP Conference Proceedings (Vol. 3377, No. 1, p. 070008). AIP Publishing LLC. (2025), October.

