# Supplementary material for: Intelligent automated essay scoring under uncertainty using type 2 neutrosophic ontologies
Source: Sci Rep. 2026 Jun 3;16:17186. doi: 10.1038/s41598-026-54596-9 (PMC13234136; doi:10.1038/s41598-026-54596-9)
Supplement: Supplementary file 1 — Supplementary Material 1 [file 41598_2026_54596_MOESM1_ESM.pdf]

README: T2NO-AES Framework Implementation

1. Overview

This supplementary material contains the complete Python source code for the **Type-2 Neutrosophic Ontology-based Automated Essay Scoring (T2NO-AES)** framework. The framework is designed to move beyond traditional automated scoring by explicitly modeling the uncertainty and ambiguity inherent in human essay evaluation using interval-valued neutrosophic logic.

2. Environment Setup

To ensure replicability, it is recommended to use a virtual environment (**Python 3.8 or higher**).

Dependencies:

Install the required standard NLP and machine learning libraries using the following command:

Bash
```
pip install numpy torch nltk spacy scikit-learn sentence-transformers
```

Resource Initialization:

The script utilizes **NLTK** and **SpaCy** for linguistic analysis. On the first run, ensure the following are downloaded:

- **NLTK:** punkt, stopwords, wordnet, averaged_perceptron_tagger.
- **SpaCy:** en_core_web_sm (for advanced NER and Dependency Parsing).

3. Implementation Modules

3.1 Preprocessing Pipeline

The preprocess module performs a multi-stage cleaning process:

- **Normalization:** Tokenization, lowercasing, and removal of punctuation/HTML/special characters.
- **Linguistic Analysis:** Stop-word elimination, lemmatization, sentence segmentation, POS tagging, and **Named Entity Recognition (NER)**.

3.2 Feature Extraction & Representation

The framework extracts features across three semantic layers:

- **Lexical:** TF-IDF weights to capture domain-specific keyword importance.
- **Contextual: Word2Vec** and **BERT** embeddings for deep semantic representation.
- **Relational:** Cosine similarity for cohesion and **Spatial Autocorrelation metrics** (Moran's I, Geary's C, Getis-Ord's G) to assess conceptual clustering.
- **Normalization:** All extracted features are normalized to the **[0, 1]** range prior to ontology mapping.

3.3 Type-2 Neutrosophic Reasoning

The code implements the formal definitions in **Section 4.1**:

- Calculates **Truth (T)**, **Indeterminacy (I)**, and **Falsity (F)** as interval-valued memberships.
- Captures the **Footprint of Uncertainty (FOU)** by defining Upper and Lower Membership Functions (UMF/LMF).

4. Execution and Validation Strategy

4.1 Training Configuration

The model utilizes linear regression with **Ridge and Lasso regularization** to prevent overfitting. To ensure deterministic results for peer review, the implementation uses **fixed random seeds**.

4.2 Validation Strategy

The system follows a **Stratified K-Fold Cross-Validation** approach. This ensures:

- Robust evaluation across diverse essay topics.
- Balanced representation of score distributions in training, validation, and testing subsets.

5. Evaluation Metrics

The framework outputs evaluation results based on standard industry metrics:

- **Quadratic Weighted Kappa (QWK):** To assess agreement with human raters.
- **Pearson Correlation Coefficient:** To measure the linear relationship between predicted and actual scores.

- **Error Metrics:** Including Mean Absolute Error (MAE) and Root Mean Square Error (RMSE).

6. Usage Instructions

1. **Dataset Preparation:** Ensure your essays are in a .csv or .json format as specified in the script's data loader.
2. **Execution:** Run the main script:

   Bash

   ```
   python T2NO_AES_Main.py
   ```

3. **Output:** The system will generate a log file containing the neutrosophic intervals for each essay and a summary of the evaluation metrics.

Source Code Documentation

```
import numpy as np
import nltk
import torch
from sklearn.feature_extraction.text import TfidfVectorizer
from sklearn.metrics.pairwise import cosine_similarity
from sentence_transformers import SentenceTransformer
from nltk.stem import WordNetLemmatizer
from nltk.corpus import stopwords
import string


# Download necessary NLTK resources

nltk.download('punkt')
nltk.download('stopwords')
nltk.download('wordnet')
nltk.download('averaged_perceptron_tagger')


class T2NO_AES_Framework:

    def __init__(self):
```

```
    self.lemmatizer = WordNetLemmatizer()
    self.stop_words = set(stopwords.words('english'))
    # Load BERT model for Step 3: Semantic Representation
    self.bert_model = SentenceTransformer('paraphrase-distilroberta-base-v1')
    self.tfidf_vectorizer = TfidfVectorizer()

# ==========================================
# STEP 1: ESSAY PREPROCESSING
# ==========================================
def preprocess(self, text):
    """Cleans and normalizes the essay text."""
    # Lowercasing & Removing Punctuation
    text = text.lower().translate(str.maketrans('', '', string.punctuation))
    # Tokenization
    tokens = nltk.word_tokenize(text)
    # Stopword removal and Lemmatization
    cleaned_tokens = [self.lemmatizer.lemmatize(w) for w in tokens if w not in self.stop_words]
    return " ".join(cleaned_tokens), cleaned_tokens
# ==========================================
# STEP 2: FEATURE EXTRACTION (Lexical/Structural)
# ==========================================
def extract_lexical_features(self, text, tokens):

    """Calculates lexical density and variety."""
    total_words = len(text.split())
    unique_words = len(set(tokens))
    # Lexical Density (Information richness)
    lexical_density = len(tokens) / total_words if total_words > 0 else 0
    # Lexical Variation
    lexical_variation = unique_words / total_words if total_words > 0 else 0
    return {"density": lexical_density, "variation": lexical_variation}

# ==========================================
# STEP 3: SEMANTIC ANALYSIS (BERT & Spatial Logic)
# ==========================================
def compute_semantic_metrics(self, essay_text, corpus):
    """Generates BERT embeddings and simulates spatial autocorrelation."""
    # BERT Embeddings
```

```
    embeddings = self.bert_model.encode([essay_text])[0]

    # TF-IDF (Global Importance)
    self.tfidf_vectorizer.fit(corpus)
    tfidf_matrix = self.tfidf_vectorizer.transform([essay_text])
    tfidf_score = np.mean(tfidf_matrix.toarray())

    # Simplified Moran's I (Global Clustering of ideas)
    # In a real scenario, this involves high-dim distance matrices
    morans_i = np.var(embeddings) / (np.mean(embeddings) + 1e-9)

    return {
        "bert_features": embeddings,
        "tfidf_relevance": tfidf_score,
        "morans_i": np.clip(morans_i, 0, 1)
    }


# ==========================================
# STEP 4: TYPE-2 NEUTROSOPHIC ONTOLOGY (T2NO)
# ==========================================
def calculate_t2_neutrosophic_score(self, feature_val, sigma=0.1, lambda_param=0.5):
    """
    Implements Section 4.1: Formal Definition.
    Maps features to [Truth, Indeterminacy, Falsity] intervals.
    """
    # 1. Truth Membership (T) as Interval [LMF, UMF]
    t_lmf = max(0, feature_val - sigma)
    t_umf = min(1, feature_val + sigma)

    # 2. Indeterminacy (I) - derived from variance/uncertainty
    i_lmf = lambda_param * (1 - feature_val)
    i_umf = min(1, i_lmf + sigma)

    # 3. Falsity (F) - Neutrosophic Complementarity
    f_lmf = 1 - t_umf
    f_umf = 1 - t_lmf

    # Final Crisp Score via Neutrosophic mapping (Truth - Falsity)
```

```
        # As per the methodology provided: Score = T - F
        avg_truth = (t_lmf + t_umf) / 2
        avg_falsity = (f_lmf + f_umf) / 2
                return avg_truth - avg_falsity
    # ==========================================
    # INTEGRATION: PIPELINE EXECUTION
    # ==========================================
    def evaluate_essay(self, essay, prompt_context_corpus):
        # Step 1
        clean_text, tokens = self.preprocess(essay)

        # Step 2
        lex_feats = self.extract_lexical_features(essay, tokens)

        # Step 3
        sem_feats = self.compute_semantic_metrics(clean_text, prompt_context_corpus)

        # Step 4: Map to T2NO (Example with Argument Clarity/Coherence)
        # We aggregate features to define "Content Quality"
        combined_feature = (lex_feats['density'] + sem_feats['morans_i'] + sem_feats['tfidf_relevance']) / 3
         final_score = self.calculate_t2_neutrosophic_score(combined_feature)
        return {
            "Lexical Density": round(lex_feats['density'], 4),
            "Semantic Coherence (Moran's I)": round(sem_feats['morans_i'], 4),
            "Final Neutrosophic Score": round(final_score, 4)
        }
# ==========================================
# EXAMPLE USAGE
# ==========================================
if __name__ == "__main__":
    aes_system = T2NO_AES_Framework()

    sample_essay = """
    Climate change is one of the most critical challenges of our time.
    Governments and individuals must act to reduce carbon emissions
    and protect the environment. Scientific evidence shows rising
    temperatures lead to melting ice caps and extreme weather.
    """
```

```
# Mock corpus for TF-IDF training
corpus = [
    "Climate change and global warming impacts.",
    "Environmental protection and carbon footprints.",
    "The role of policy in climate mitigation."
]
results = aes_system.evaluate_essay(sample_essay, corpus)
print("--- T2NO-AES Evaluation Results ---")
for key, val in results.items():
    print(f"{key}: {val}")
```
